# Therapeutic Efficacy of *Lavandula dentata*’s Oil and Ethanol Extract in Regulation of the Neuroinflammation, Histopathological Alterations, Oxidative Stress, and Restoring Balance Treg Cells Expressing FoxP3+ in a Rat Model of Epilepsy

**DOI:** 10.3390/ph18010035

**Published:** 2024-12-31

**Authors:** Aziza Antar, Eman S. Abdel-Rehiem, Areej A. Al-Khalaf, Abdelaziz S. A. Abuelsaad, Mohamed Abdel-Gabbar, Gaber M. G. Shehab, Ayman M. Abdel-Aziz

**Affiliations:** 1 Biochemistry Department, Faculty of Science, Beni-Suef University, Beni-Suef 62521, Egypt; gomana777@yahoo.com (A.A.); hhmgabar@yahoo.com (M.A.-G.); 2 Molecular Physiology Division, Zoology Department, Faculty of Science, Beni-Suef University, Beni-Suef 62521, Egypt; ema4salah@yahoo.com; 3 Biology Department, College of Science, Princess Nourah Bint Abdulrahman University, P.O. Box 84428, Riyadh 11671, Saudi Arabia; aaalkhalaf@pnu.edu.sa; 4 Immunology Division, Zoology Department, Faculty of Science, Beni-Suef University, Beni-Suef 62521, Egypt; 5 Department of Biochemistry, College of Medicine, Taif University, P.O. Box 11099, Taif 21944, Saudi Arabia; g.shehab@hotmail.com; 6 Zoology Department, Faculty of Science, Fayoum University, Fayoum 63514, Egypt; ama25@fayoum.edu.eg

**Keywords:** epilepsy, *Lavandula dentata*, neurotransmitters, electrolytes, oxidative stress, hippocampus, FOXP3 and CTLA-4

## Abstract

**Background/Objectives**: Despite the availability of antiepileptic drugs (AEDs) that can manage seizures, they often come with cognitive side effects. Furthermore, the role of oxidative stress and neuroinflammatory responses in epilepsy and the limitations of current AEDs necessitate exploring alternative therapeutic options. Medicinal plants, e.g., *Lavandula dentata* L., are rich in phenolic compounds and may provide neuroprotective and anti-inflammatory benefits. However, limited research evaluates their effectiveness in modulating neuroinflammation and histopathological changes in epilepsy models. Therefore, the current study hypothesized that treating *Lavandula dentata* L. extract or essential oils may reduce neuroinflammatory responses and mitigate histopathological changes in the brain, providing a natural alternative or adjunct therapy for epilepsy management. **Methods**: Five groups of male Wistar rats were used: control, pilocarpine-treated epileptic, valproic acid (VPA-treated epileptic), *L. dentata* extract, and essential oils. Numerous electrolyte levels, monoamine levels, neurotransmitter levels, and the mRNA expression of specific gate channel subtypes were evaluated in homogenate brain tissue. Additionally, histological changes in various brain regions were investigated. **Results**: The investigation revealed that the extract and essential oils obtained from *L. dentata* L. exhibited the ability to improve the modulation of electrolytes and ions across voltage- and ligand-gated ion channels. Furthermore, it was revealed that they could decrease neuronal excitability by facilitating repolarization. Moreover, *L. dentata’s* oil and ethanol extract re-balances T-reg/Th-17 cytokines, restoring the pro/anti-inflammatory cytokines and Treg markers, e.g., FOXP3 and CTLA-4, to their normal level. **Conclusions**: The present work confirms that the extract and essential oils of *L. dentata* L. have different activities to ameliorate the progression of histopathological alterations. Therefore, when used in conjunction with other AEDs, the extract and essential oils of *L. dentata* can slow the progression of epileptogenesis.

## 1. Introduction

Epilepsy is among the most prevalent and incapacitating neurological conditions [1]. With a high rate of pharmaco-resistance, a variety of comorbidities (including progressive cognitive and behavioral disorders), and increased mortality from direct (e.g., sudden unexpected death in epilepsy, accidents, drowning) or indirect effects of seizures and therapies, it is a prevalent disorder that impacts more than 70 million people globally. Considerable investigation utilizing animal models and human subjects has yielded restricted knowledge regarding the mechanisms that underlie epileptogenesis and seizures; furthermore, these findings have not resulted in substantial reductions in pharmaco-resistance, morbidities, or mortality [2].

Epilepsy is characterized by recurrent, unprovoked episodes of seizures due to a disorder in the synchronized activity of neurons [3]. Specific brain regions may experience a neurochemical imbalance due to uncontrolled status epilepticus (SE) [4]. Excessive production of free radicals has been linked to the development of neuronal hyperactivity and/or excitotoxicity [5]. The term “epilepsy” describes a group of illnesses characterized by recurrent spontaneous seizures that appear to be the consequence of complicated processes involving numerous neurotransmitter systems, including glutamatergic, cholinergic, and GABAergic systems [6]. It is brought on by sporadic, highly coordinated electric neuronal bursts that affect the brain’s tiny or large neuronal networks, impairing mental function, movement, awareness, or a combination of these symptoms [7].

Several novel antiepileptic medicines (AEDs) have been approved for the treatment of seizures [8]. The prevention of seizures is the primary goal of AED medication. As a result, once patients have achieved a seizure-free state, avoiding even a single seizure revolution occurrence is critical. Individuals with epilepsy, particularly those who are seizure-free, require consistency and certainty in their AED prescription to avoid breakthrough seizures [9]. About 25% of epileptic individuals have drug-resistant epilepsy [10]. Their weak control over epileptic seizures (ESs) puts them at risk for early mortality, trauma, and psychological problems, and it also lowers their quality of life. Four percent of adult cases of drug-resistant epilepsy go into remission each year; rates are higher in children. However, recurrence of ES is possible at any time. Therefore, to start the pre-surgical evaluation process, it is essential to identify patients with drug-resistant epilepsy and decide on other prospective therapeutic alternatives in specialized units or centers [10].

While antiepileptic medicine has been shown to enhance cognitive function, it is essential to note that it is also associated with a significant incidence of cognitive adverse effects. Mental side effects are therapeutically substantial in that they may affect treatment adherence, long-term memory retention, various aspects of daily functioning, and overall health-related quality of life. While antiepileptic medicine has been shown to enhance cognitive function, it is essential to note that it is also associated with a significant incidence of cognitive adverse effects. Mental side effects are therapeutically substantial in that they may affect treatment adherence, long-term memory retention, various aspects of daily functioning, and overall health-related quality of life. The present analysis assesses and evaluates the subject matter.

One of the key areas of research right now is looking for new antiepileptic drugs that can also protect neurons. Using plants and plant extracts to treat illness is an example of a therapeutic method. According to the World Health Organization (WHO), traditional techniques, primarily using plants, provide healthcare for approximately 75% of the world’s population [11]. Therefore, the folk medicines that were turned into natural medicines have been essential in the progress of modern drugs. It can be utilized to find an alternate source of AEDs with innovative structures and enhanced safety and effectiveness profiles. It can also be combined with low-dosage medications [12]. Medicinal plants are good at treating various illnesses because they contain several active chemicals, mainly phenolic products like flavonoids, phenylpropanoids, stilbenes, and others. Several substances have been shown to have antioxidant qualities. They might help protect against the harmful effects of reactive oxygen species [13].

The genus *Lavandula* belongs to the Labiatae family (Lamiaceae). Lavandula species can be found all around the Mediterranean region and are grown in France, Spain, and Italy. The perfume, cosmetic, flavoring, and pharmaceutical industries all depend on the essential oil of Lavandula species. Lavender oils (LOs) are primarily used as raw materials in industrial perfume and fragrance products, with most lavender oils used this way [14]. Malcolm and Tallian [15] discovered that LO oral solutions help people feel calm and relaxed more quickly than first-line anxiety medications such benzodiazepines and serotonin reuptake inhibitors. As Sayorwan, Siripornpanich [16] say, this calm effect is caused by LO’s impact on the autonomous nervous system (ANS). Essential oils are attracting more attention lately as a way to treat several nerve illnesses. The biological effect of *L. dentata* oil could partly be explained by an inhibitory action on NMDA glutamate receptors [17]. Therefore, it was found that LEO can improve learning and memory in Alzheimer’s Disease [18] via AMPA glutamate receptors.

*Lavandula dentata* has recently had a long history of traditional medicinal use in medical treatments, adding flavor to recipes or infusions and culinary applications. *L. dentata* has been reported to be used for food therapy, intestinal issues, respiratory infections, and cough problems. Its essential oils are used in aromatherapy for calming and relaxing properties [19,20]. Furthermore, *L. dentata* is recognized for its anti-arthritic properties, which help reduce arthritis and inflammation [20]. On the other hand, *L. dentata* essential oil is known for its antibacterial, diuretic, and healing properties. It is commonly used in infusion or decoctions to treat cold symptoms and infections such as renal coli [21].

However, until today, there were no reports on the neuroinflammatory, histopathological alterations, oxidative stress, inflammatory cytokines, and FoxP3+ Treg cell activities of *Lavandula dentata* L. in the case of an epileptic rat model. Therefore, the novel objectives of the current study were to evaluate the antioxidant and immunomodulatory properties of the ethanolic extract and essential oils derived from *Lavandula dentata* L in a rat model and study its ability to modulate FOXP3+ regulatory T cells and restore immune balance, which was not previously documented.

## 2. Results

### 2.1. Electrolytes in Sera and Tissues

The brain tissue of the epilepsy group (Table 1) had a notable decrease in sodium (Na^+^) content, which recorded a decline of 128.89 ± 0.46 mmol/L compared to the control group, which had a higher sodium concentration in tissue. In contrast, the hippocampus homogenate tissue of the PILO-induced epilepsy group (EP) exhibited notable hypernatremia (*p* < 0.05). Moreover, a statistically significant increase (*p* < 0.05) in Na^+^ was observed in the serum of animals treated with valproic acid (EP-VPA) in comparison to the epileptic group. In contrast, the level of sodium ions (Na^+^) in the hippocampal homogenate tissue valproic acid-treated group (EP-VPA) showed a statistically significant decrease (*p* < 0.05) compared to EP. Furthermore, for oral administration with *L. dentata* oil (EP-LO) or *L. dentata* extract (EP-LX), a statistically significant elevation (*p* < 0.05) in serum sodium (Na^+^) levels comparable with the EP group (128.89 ± 0.46 mM/mL) was reported, in contrast to a substantial decrease in the homogenate tissue of the EP-LO-treated group, parallel to a nonsignificant reduction (*p* < 0.05) in EP-LX (Table 1). Moreover, the data presented in Table 1 indicates statistically significant hyperkalemia in EP’s sera (7.13 ± 0.28 mmol/L), in contrast to other groups, e.g., EP-VPA, EP-LO, and EP-LX, which exhibited low potassium contents. In addition, the levels of potassium ions (K^+^) in the homogenized tissues of the EP group showed a significant decrease (*p* < 0.05) as compared to the control and EP-VPA groups. On the other hand, both EP-LO and EP-LX treatments exhibit more K^+^ levels in homogenate tissues (Table 1).

Regarding ionized calcium (Ca^++^) in the sera, it was found that there was a statistically significant decrease (*p* < 0.001) in animals with EP compared to the control and EP-VPA groups. In addition, it was observed that both the EP-LO- and EP-LX-treated groups had a noteworthy elevation (*p* > 0.05) in the concentration of serum Ca^++^ (0.76 ± 0.12 and 0.99 ± 0.08 mM/mL, respectively). On the other hand, a statistically significant rise in hypercalcemia (*p* < 0.001) was noted in the hippocampus homogenate tissue of the EP group (0.60 ± 0.02 mmol/L Tissue) in comparison to the control group (0.30 ± 0.06 mmol/L Tissue). EP-VPA, EP-LO, and EP-LX groups exhibited lower levels of calcemia in contrast to the diseased group (Table 1). Moreover, Table 1 revealed a highly significant hyperchloremia (115.57 ± 2.77 mM/mL) in the EP group’s serum and a highly substantial hypochloremia (73.08 ± 6.45 mM/mL) in their hippocampal homogenate tissues. Furthermore, findings showed that Cl-w was considerably recovered and retained to normal (*p* > 0.001) in EP-VPAs’ serum or hippocampus homogenate tissues. Additionally, the hippocampal homogenate tissues of EP-LO and EP-LX groups demonstrated a highly significant elevation (*p* > 0.001), and the Cl^−^ levels in their serum were significantly (*p* > 0.001) ameliorated to the control level.

Regarding the gene expression of some gates, the mRNA expression of SCN1A (Na^+^) exhibited a statistically significant increase (*p* < 0.001) in the epileptic group EP (1.44 ± 0.01 versus 1.01 ± 0.01). Conversely, VPA treatment recorded a relative expression of 0.79 ± 0.03 and demonstrated a significant decrease (*p* < 0.05). Additionally, there was a substantial (*p* < 0.05) drop in the expression of SCN1A (Na^+^) mRNA between *L. dentata* oil (EP-LO) and *L. dentata* extract (EP-LX), with respective values of 0.81 ± 0.05 and 0.78 ± 0.05 (Table 2).

Expression of the potassium gate channel gene KCNJ2 (K+) in the EP group recorded a significant decrease (*p* < 0.001) (0.36 ± 0.05), while treatment with VPA, LO, LX exhibited a significant increase (*p* < 0.05) with reported values of 0.62 ± 0.05, 0.65 ± 0.10, and 0.58 ± 0.08, respectively) (Table 2). Moreover, the pilocarpine induction group exhibited the maximum mRNA expression (*p* < 0.001) of CACNA1S (Cav1.1) (7.07 ± 0.40) compared to controls (1.01 ± 0.01). Also, EP-VPA, EP-LO, and EP-LX groups showed a significant decrease (*p* < 0.05) and recorded the lowest expression (3.17 ± 0.35, 2.87 ± 0.33, and 2.54 ± 0.60, respectively) (Table 2).

The GABA receptor functions as a ligand-gated ion channel. The findings of this study indicate that the expression of CLCNC mRNA in the EP group decreased significantly (*p* < 0.001) when compared to the group serving as the control (1.03 ± 0.02). Furthermore, it was observed that the mRNA expression of CLCNC was considerably more significant in the EP-VPA, EP-LO, and EP-LX groups (*p* < 0.05): 0.62 ± 0.03, 0.77 ± 0.08, and 0.56 ± 0.06, versus the EP (0.14 ± 0.03). (Table 2). Furthermore, the present data revealed a significant increase (*p* < 0.001) in NMDA mRNA gene expression in EP (7.71 ± 1.63) compared to controls (1.01 ± 0.01). On the other hand, there was a substantial decrease (*p* < 0.05) in the gene expression of mRNA of NMDA in the EP-VPA, EP-LO, and EP-LX groups in comparison to the EP group, but it was still significantly higher than that of healthy condition (Table 2).

In terms of changes in neurotransmitters, Table 3 demonstrates that the concentration of L-DOPA in hippocampus homogenate tissue from the EP group was markedly elevated (36.40 ± 2.352 Ug/protein tissue) (*p* < 0.001) and was greater than that of the controls. Conversely, the hippocampal DOPA level in the VPA-treated group was found to be significantly restored, close to the control (*p* < 0.05). Additionally, the tissue’s DOPA level was decreased significantly (*p* < 0.05) after oral administration of *L. dentata* oil or extract. Moreover, the present data recorded that EP had the highest (*p* < 0.001) increase in epinephrine concentration (134.67 ± 5.860 pg/mg protein tissue) in contrast to the control group (78.63 ± 2.143 pg/mg protein tissue) (Table 3). Moreover, there was a significant reduction (*p* < 0.05) in all treated groups, as they EP-VPA, EP-LO, and EP-LX recorded 91.67 ± 2.410, 92.83 ± 1.849, and 93.83 ± 2.167 pg/mg protein tissue, respectively.

Concerning norepinephrine (NE), it was noticed that EP showed a statistically significant increased level (*p* < 0.001) with a mean value of 213.10 ± 8.365 pg/mg protein tissue (Table 3) as compared to the control group (108.67 ± 3.299 pg/mg protein tissue). Significantly, its level in the hippocampal homogenate tissue of both EP-VPA and EP-LX groups was lower (*p* < 0.05) than the EP group, while EP-LO showed a highly significant reduction in NE level compared to EP (133.03 ± 1.590 pg/mg protein tissue).

Furthermore, the EP’s γ-aminobutyric acid (GABA) level was significantly decreased (19.13 ± 1.20 pg/mg protein tissue) compared to controls (57.23 ± 0.69 pg/mg protein tissue) (Table 3). On the other hand, GABA concentration inside the hippocampal tissue was significantly higher (*p* < 0.05) in the EP-VPA, EP-LX, and EP-LO groups and ameliorated to normal levels.

Regarding brain glutamate in the epileptic group (EP), it recorded (*p* > 0.001) 24.10 ± 2.065 ng/mg protein tissue, i.e., significantly higher than that of the control group. EP-VPA exhibited a highly significant reduction (*p* > 0.001) in hippocampal glutamate content (7.50 ± 1.273 ng/mg protein tissue) compared to EP, suggesting that EP-VPA levels are closer to those observed in the control group. Additionally, oral treatment with *L. dentata* oil (EP-LO) or extract (EP-LX) demonstrated a more pronounced reduction (*p* > 0.001) in glutamate levels. Additionally, the current investigation demonstrated a statistically significant (*p* < 0.001) reduction in the brain acetylcholinesterase (AChE) level in EP (38.43 ± 3.896 U/mg protein tissue) in contrast to the control group (95.43 ± 3.552 U/mg protein Tumor). Conversely, the AChE level in the hippocampus tissue (73.10 ± 4.351 U/mg) increased significantly (*p* < 0.05) according to EP-VPA. Based on the information available, oral administration of *L. dentata* oil (EP-LO) or extract (EP-LX) increased the AChE concentration of homogenate hippocampus tissue by a significant (*p* < 0.05) amount, with values of 70.87 ± 4.535 and 89.57 ± 4.535 U/gm protein tissue, respectively (Table 3).

### 2.2. Evaluation of Oxidative Stress Biomarkers in the Hippocampus

**Figure 1:**
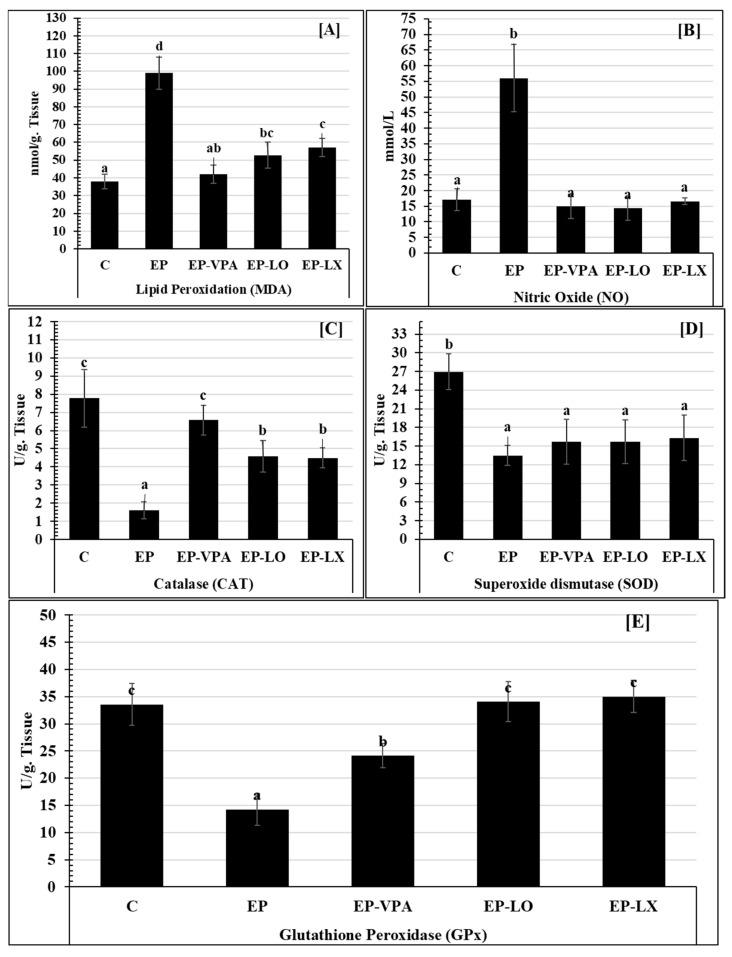
Changes in prooxidant markers in hippocampal homogenate tissues: panel (**A**) lipid peroxidation and (**B**) nitric oxide; (**C**) catalase, (**D**) superoxide dismutase, and (**E**) glutathione peroxidase of different groups: control (C), epileptic (EP), valproic acid-treated (EP-VPA), *L. dentata* oil-treated (EP-LO), and *L. dentata* extract-treated (EP-LX). The data were analyzed using the Tukey–Kramer method for post hoc analysis to compare various groups with each other. Subset for alpha = 0.05 and the means for groups in homogeneous subsets are displayed. All values are represented as Mean ± Std and n = 8 animals/group. Means within the same parameter and not sharing a common superscript symbol(s) differ significantly at *p* < 0.05.

Lipid peroxidation is articulated as malonaldehyde (MDA) concentration and was measured as a biomarker of oxidative stress in brain tissues. When comparing the hippocampal LPO concentration (99.00 ± 9.00 nmol/g. tissues) of the pilocarpine group to that of the control group (C) (38.00 ± 4.04 nmol/g. tissues), a substantial rise (*p* > 0.001) was observed (Figure 1A). Conversely, compared to the EP group (99.0 ± 9.0 nmol/g. tissues), the valproic acid-treated (EP-VPA) group had a substantial drop in LPO (*p* > 0.001) (42.00 ± 5.20 nmol/g. tissues). In comparison to the diseased group EP (99.00 ± 9.00 nmol/g. tissues), the oral treatment with either *Lavandula dentata* oil (EP-LO) or *L. dentata* extract (EP-LX) resulted in a significant (*p* > 0.001) decrease in the LPO levels of the hippocampal homogenate tissue, with recorded values of 52.80 ± 7.27 and 57.00 ± 5.20 nmol/g. tissues, respectively.

In synapses, nitric oxide (NO) is thought to function as a retrograde neurotransmitter that facilitates brain blood flow and is crucial for intracellular signaling. In comparison to the control groups (17.08 ± 3.47 mmole/L), Figure 1B demonstrates that the NO recorded tremendously significant increase (*p* < 0.001) changes in the EP group of 56.02 ± 10.75 mmole/L. The current findings showed that the levels of NO in hippocampus homogenate tissue were significantly lower (*p* > 0.001) in EP-VPA (15.00 ± 3.86 mmole/L) than in EP (56.02 ± 10.75 mmole/L). According to the current findings, when compared to the non-treated group (EP), oral treatment with either *L. dentata* oil (EP-LO) or *L. dentata* extract (EP-LX) resulted in a significant (*p* > 0.001) drop in NO levels, measuring 14.32 ± 3.80 and 16.60 ± 1.16 mmole/L, respectively.

In the epileptic group (EP), the activity of the neural catalase (CAT) enzyme decreased significantly (*p* > 0.001) to 1.60 ± 0.46 U/g. tissue (Figure 1C), in comparison to the controls. On the other hand, the EP-VPA group recorded a significantly ameliorated level (*p* > 0.001) of CAT activity (6.58 ± 0.82 U/g. tissue), which appears to approach the control level. In addition, the present data showed that the oral treatment with either *L. dentata* oil (EP-LO) or extract (EP-LX) showed more significant (*p* > 0.001) release of CAT levels, which was drawing near the control level and recorded 4.58 ± 0.86 and 4.49 ± 0.55 U/g. tissue, respectively.

Unexpectedly, the current biochemical analysis revealed a substantial reduction (*p* > 0.001) in the SOD concentration (Figure 1D) across all groups, including EP, EP-VPA, *L. dentata* oil (EP-LO), and extract (EP-LX), whereas they recorded 13.47 ± 1.60, 15.71 ± 3.61, 15.70 ± 3.54, and 16.29 ± 3.66 U/g. tissue, respectively, comparable with the control group (26.94 ± 2.90 U/g. tissue). Moreover, all diseased or treated groups showed non-significant variations between each other (*p* > 0.05).

The results of the current investigation (Figure 1E) demonstrated that pilocarpine significantly reduced glutathione peroxidase (GPX) to 14.25 ± 2.92 U/g. tissue, as compared to the controls (*p* > 0.001). Also, there was a significant change (*p* > 0.001) between the EP-VPA (24.10 ± 2.23 U/g. tissue) and EP groups. Furthermore, compared to EP (14.25 ± 2.92 U/g. tissue), the brain tissues of epileptic rats treated with either *L. dentata* oil (EP-LO) or extract (EP-LX) demonstrated a considerable increase (*p* > 0.001) in GPX contents (34.04 ± 3.67 and 35.02 ± 2.92 U/g. tissue, respectively). They almost approached the control level (33.56 ± 3.86 U/g. tissue) with a non-significant difference between the three groups (C, EP-LO, and EP-LX).

**Figure 2:**
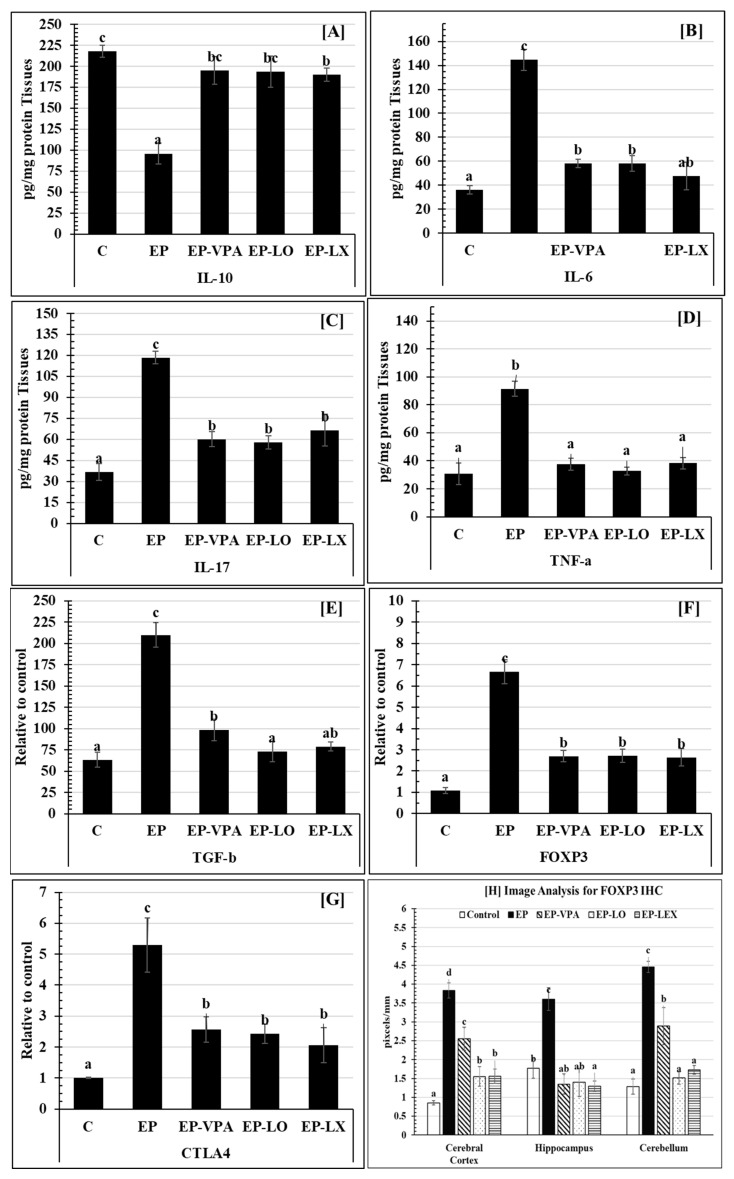
Changes in prooxidant markers in hippocampal homogenate tissues: panel (**A**) IL-10; (**B**) IL-6; (**C**) IL-17, (**D**) TNF-a; (**E**) TGF-b; (**F**) FOXP3 expression; (**G**) CTLA-4 expression; and (**H**) IHC quantification image analysis for the stained FOXP+ cells in different regions of hippocampal sections of different groups: control (C), epileptic (EP), valproic acid-treated (EP-VPA), *L. dentata* oil-treated (EP-LO) and *L. dentata* extract-treated (EP-LX). The data were analyzed using the Tukey–Kramer method for post hoc analysis to compare various groups with each other. Subset for alpha = 0.05 and the means for groups in homogeneous subsets are displayed. All values are represented as Mean ± Std and n = 8 animals/group. Means within the same parameter and not sharing a common superscript symbol(s) differ significantly at *p* < 0.05.

Evaluation of the inflammatory cytokines (Figure 2) revealed that when compared to the healthy group (C) (217.90 ± 6.96 pg/mg protein tissue), the hippocampal tissue of rats administered pilocarpine showed a significant drop (*p* > 0.001) in IL-10 cytokine (Figure 2A) (95.90 ± 12.53 pg/mg protein tissue) compared to the EP-VPA group; it was observed that the hippocampus IL-10 content increased significantly (*p* > 0.05) to 195.10 ± 16.45 pg/mg protein tissue. The results of the present study indicated that the application of *Lavandula dentata* oil (EP-LO) or extract (EP-LX) via the oral route led to a statistically significant elevation (*p* > 0.05) in the IL-10 concentrations of hippocampal homogenate tissue (193.80 ± 18.60 and 190.03 ± 8.15 pg/mg protein tissue, respectively). These levels were similar to those observed in the EP of the diseased group (95.90 ± 12.53 pg/mg protein tissue).

A pleiotropic cytokine, IL-6, is released in reaction to infections and tissue injury. As illustrated in Figure 2B, the EP group recorded a more considerable significant rise (*p* < 0.001) in IL-6 (144.60 ± 8.71 pg/mg protein tissue) compared to the control groups, which recorded 36.07 ± 3.62 pg/mg protein tissue. The results of this study demonstrated a significant (*p* > 0.05) decrease in IL-6 levels in hippocampal homogenate tissue between EP-VPA (58.33 ± 3.50 pg/mg protein tissue) and EP (144.60 ± 8.71 pg/mg protein tissue). The oral treatment with either *L. dentata* oil (EP-LO) or *L. dentata* extract (EP-LX) resulted in a significant (*p* > 0.05) decrease in IL-6 levels, recording 58.20 ± 6.46 and 47.50 ± 11.55 pg/mg protein tissue, respectively, when compared to the non-treated group (EP) (144.60 ± 8.71 pg/mg protein tissue).

The IL-17 level in the hippocampal homogenate tissue (Figure 2C) of the non-treated epileptic group (EP) was significantly elevated (*p* < 0.001) compared to the control group (36.70 ± 6.03 pg/mg protein tissue) (118.37 ± 4.42 pg/mg protein tissue). Conversely, the oral treatment EP-VPA, EP-LO, and EP-LX groups exhibited noticeably reduced IL-17 (*p* > 0.05) levels of 60.00 ± 5.43, 57.77 ± 4.84, and 66.40 ± 11.34 pg/mg protein tissue, in that order.

In comparison to the control groups (30.60 ± 7.78 pg/mg protein tissue), the EP group’s TNF-α recorded a substantial rise (*p* < 0.05) of 91.40 ± 5.47 pg/mg protein tissue, as shown in Figure 2D. In comparison to the EP group, the current results demonstrated a significant (*p* > 0.05) decrease in TNF-α level in hippocampus homogenate tissue in EP-VPA (37.57 ± 4.41 pg/mg protein tissue). Furthermore, the oral treatment with either EP-LO or EP-LX ameliorated the level of TNF-α and observed a significant decrease of 32.80 ± 2.72, 38.37 ± 4.15 pg/mg protein tissue (*p* > 0.05), respectively.

The transforming growth factor-beta (TGF-b) was found to be significantly elevated (*p* > 0.001) by pilocarpine (Figure 2E), recording 210 ± 14.46 pg/mg protein tissue compared to 63.33 ± 8.85 pg/mg protein tissue in the control group. Additionally, the EP group and EP-VPA (98.37 ± 12.58 pg/mg protein tissue) significantly decreased (*p* > 0.05). Instead, brain tissues from rats with epilepsy that were given *L. dentata* oil (EP-LO) or extract (EP-LX) showed a significant (*p* < 0.001) decrease in TGF-in EP-LO (73.00 ± 12.11 pg/mg protein tissue) and an important (*p* < 0.05) decrease in EP-LX (78.83 ± 5.42 pg/mg protein tissue). Therefore, both treatments were higher than the protein tissue control level (63.33 ± 8.85 pg/mg).

**Figure 3:**
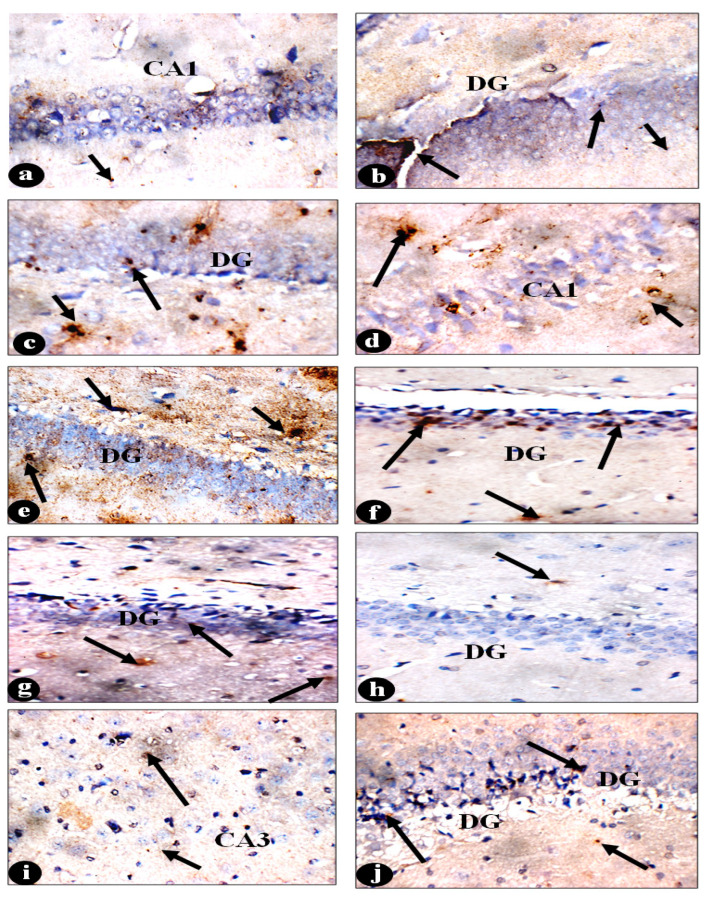
Immunohistochemistry photographs (Formalin/PFA-fixed paraffin-embedded sections) of rat brain tissue staining anti-FOXP3 using antibody CAT # ab75763 with final conc: 1/100, DAB staining (in brown) and hematoxylin QS as a counterstain (in blue). Photographs with black arrows showed positive FOXP3-bearing cells in brain tissues from different groups with positive cells for FOXP3 (arrow, ×400); (**a**,b) control; (**c**,**d**) epileptic DG’s brain; (**e**,**f**) valproic acid-treated (EP-VPA)’s DG; (**g**,**h**) DG region of sections from *L. dentata* oil-treated (EP-LO), and (**i**,**j**) sections from *L. dentata* extract-treated (EP-LX). The arrow indicates positive FOXP3+ cells.

The regulatory T (Treg) cell, mainly in charge of producing suppressive effects, depends on the Fork-head box P3 (FOXP3) protein to control gene expression. This investigation found that the EP group’s FOXP3 gene expression was significantly higher than that of the control group (*p* < 0.001). As shown in Figure 2F, the mean expression level in the EP group was 6.67 ± 0.55, while it was 1.08 ± 0.14 in the control group. On the other hand, FOXP3 mRNA gene expression was statistically significantly reduced (*p* < 0.05) in the EP-VPA group, with a mean value of 2.70 ± 0.26. Additionally, FOXP3 expression recorded the lowest level in the EP-LO and EP-LX groups (2.71 ± 0.31 and 2.64 ± 0.41, respectively) (Figure 2F). A confirmational quantification image analysis was performed for the immunohistochemistry staining of Formalin/PFA-fixed paraffin-embedded sections of rat brain tissue staining anti-FOXP3 using antibody CAT # ab75763 with final conc: 1/100, DAB staining (in brown) and hematoxylin QS as a counterstain (in blue). Image analysis of IHC-FOXP3 (Figure 2H) confirmed the re-balance of its gene expression in brain regions, whereas the quantity of the FOXP+-positive cells was increased in epileptic brain regions and recorded 3.83 ± 0.19, 3.60 ± 0.30, and 4.45 ± 0.15 in the cerebral cortex, hippocampus, and cerebellum, respectively. Significantly, LO and LX treatments showed re-balance (*p* < 0.0001) in the number of IHC-FOXP3-positive cells in all investigated regions (Figure 2H and Figure 3).

Furthermore, the data show that the levels of the messenger RNA (mRNA) for cytotoxic T-lymphocyte-associated protein 4 (CTLA4) (Figure 2G) increased statistically significantly (*p* < 0.001). It was discovered that the experimental group (EP) had 5.30 ± 0.87 CTLA4 expression, whereas the control group had 1.01 ± 0.01 expression. The gene expression of CTLA4 mRNA was significantly reduced (*p* < 0.05) in the EP-VPA, EP-LO, and EP-LX groups compared to the EP group (5.30 ± 0.87), with values of 2.57 ± 0.41, 2.43 ± 0.31, and 2.07 ± 0.57, respectively.

### 2.3. Histopathological Alterations (H&E)

**Figure 4:**
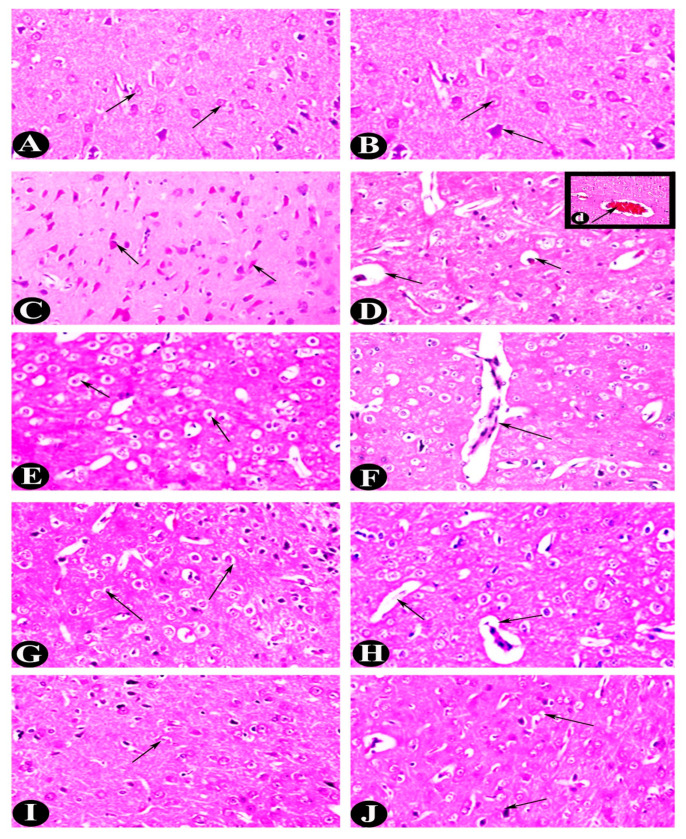
Photographs for histopathological examination (H&E ×400) for cerebral cortex from different groups, whereas (**A**,**B**) H&E showed control rats’ cerebral cortex showed a normal histological structure with no major pathological changes. (**C**,**D**) section of epileptic cerebral cortex was characterized by dark staining and voids between per-cellular haloes; in addition, a significant perivascular oedema and congestion in the cerebral blood vessels with focal hemorrhagic regions was recorded (d panel). The nuclear chromatin of some of these eosinophilic apoptotic entities was noticeably condensed and clumped. (**E**,**F**) H&E sections of EP-VPA-treated cerebral cortex with neuronal cell degeneration, which manifested as shrunken, darkly stained cells with per-cellular haloes. A small amount of neuronophagia, perivascular oedema, gliosis, apoptotic bodies, and satelitosis were also observed, scoring (2). (**G**,**H**) H&E sections of EP-LO-treated cerebral cortex with small number of nuclear pyknosis-associated deteriorated neuronal cells. In addition, perivascular oedema, neuronophagia, gliosis, and satelitosis were found. Compared to the group that did not receive treatment, there were fewer apoptotic eosinophilic neurons. Reduced, condensed, and clumped nuclear chromatin was visible in the red-stained neurons, and oligodendrocytes were present in large numbers around the nuclear chromatin (2). The nuclear chromatin of some of these eosinophilic apoptotic entities was noticeably condensed and clumped. (**I**,**J**) H&E sections of EP-LX-treated cerebral cortex exhibited normal histological structure with no notable changes, and the neuronal cells had prominent nucleoli and round nuclei surrounded by a sparse layer of basophilic cytoplasm score (0).

Regarding the light microscope study, the control rats’ cerebral cortex showed a standard histological structure with no significant pathological changes. On the other hand, the cerebral cortex comprises several layers of neuronal cells arranged in microscopic blood veins with no clear borders between them. The nuclei of the neuronal cells were spherical or oval, and surrounded by a sparse layer of basophilic cytoplasm score (0) by prominent nucleoli (Figure 4A,B). In contrast, neuronal degeneration brought on by epilepsy induction was characterized by dark staining and voids between per-cellular haloes. Neuronophagia and gliosis were also seen. Notably, neuronal cell apoptosis manifested as eosinophilic masses linked to steatosis and localized gliosis. The nuclear chromatin of some of these eosinophilic apoptotic entities was noticeably condensed and clumped score (3). Figure 4C,D showed significant perivascular edema and congestion in the cerebral blood vessels with focal hemorrhagic regions.

Furthermore, H&E sections of the adult male albino rat treated with VPA in the cerebral cortex revealed neuronal cell degeneration, manifested as shrunken, darkly stained cells with per-cellular haloes. A small amount of neuronophagia, perivascular edema, gliosis, apoptotic bodies, and steatosis was also observed, scoring (2) (Figure 4E,F). Some sections of the cerebral cortex treated with LO exhibited a small number of nuclear pyknosis-associated deteriorated neuronal cells. In addition, perivascular edema, neuronophagia, gliosis, and steatosis were found. There were fewer apoptotic eosinophilic neurons than the group that did not receive treatment. Reduced, condensed, and clumped nuclear chromatin was visible in the red-stained neurons, and oligodendrocytes were present in large numbers around the nuclear chromatin (2) (Figure 4G,H). The administration of LX reduces the various histological changes. The cerebral cortex exhibited standard histological structure with no notable changes, and the neuronal cells had prominent nucleoli and round nuclei surrounded by a sparse layer of basophilic cytoplasm score (0) (Figure 4I,J).

**Figure 5:**
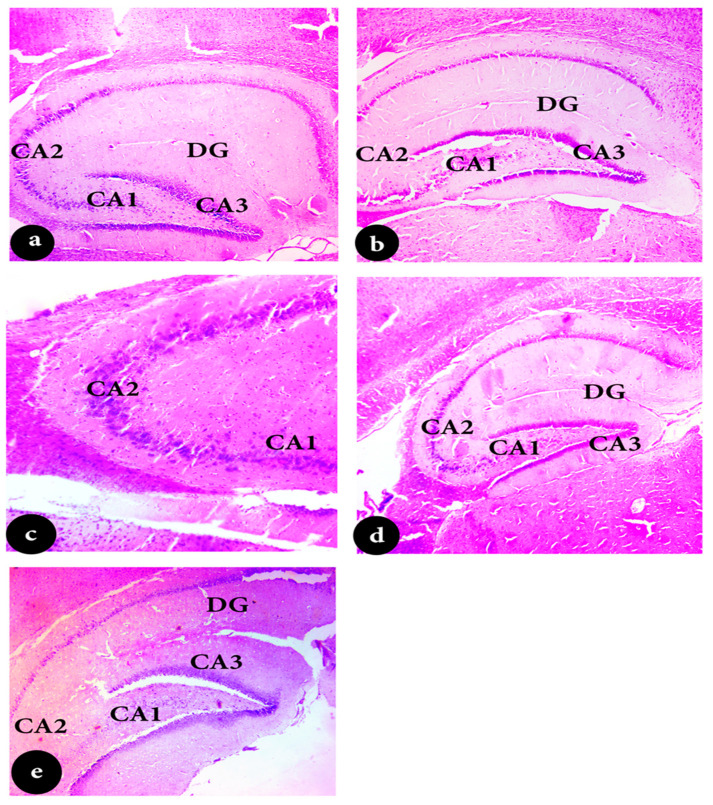
Photographs for histopathological examination (H&E) for hippocampus from different groups, whereas (**a**) section from control hippocampus showing no pathological changes (×400). (**b**) Section from epileptic rats, showing hippocampal sclerosis by gliosis and focal neuronal loss, seen mainly in CA1 level of the hippocampus (×400). (**c**) Section from valproic acid-treated (EP-VPA), which shows moderate cellular disorganization and shrinkage in size of pyramidal cells, with darkened nuclei (×400). (**d**) Section from *L. dentata* oil-treated (EP-LO), whereas no pathological changes were identified (×400). (**e**) Section from *L. dentata* extract-treated (EP-LX), which shows focal neuronal loss without gliosis (×400).

The hippocampus slices from the control animals showed layers of compact granular cells with black nuclei, characteristic of their histological structure. In the molecular layer, glial and pyramidal cells were both observable. Upon high-power examination of the hippocampus region, three layers were typically noticeable: the molecular, pyramidal, and polymorphic layers (Figure 5a). In contrast, the hippocampal portions of the EP group showed atrophy, cellular disarray, and a decrease in the number of large pyramidal cells with darker nuclei. Significant vacuolations were also visible in granular cell layers (Figure 5b). After subjecting the cells to VPA treatment, the size of the pyramidal cells shrank, and their nuclei darkened (Figure 5c; H&E ×400). Additionally, hippocampus slices from EP-LO patients showed no pathological alterations in cellular organization, except for a little reduction in pyramidal cell size and darker nuclei (Figure 5d; H&E ×400). Furthermore, EP-LX therapy demonstrated that the hippocampus retained its typical histological configuration of granular cells with black nuclei and the presence of average glial cells and pyramidal cells in the molecular layer (Figure 5e; H&E ×400).

### 2.4. Image Analysis

Image analysis quantification for histopathological alterations in the cerebral cortex, which revealed variations in all H&E photographs, was analyzed (six fields/rat/group) using the software ImageJ 1.52e.

All possible quantifications for the image analysis (Table 4) were done for the studied layers, and the highest average or normal cerebral neurons were recorded in the control group (25.50 ± 2.74). At the same time, EP was significantly reduced (13.333 ± 2.25), likely due to PILO’s negative impact. On the other hand, all treatments modulated and significantly (*p* < 0.05) recovered the normal cerebral neurons close to the normal count. Enumeration of the degenerative neurons revealed that control of the histopathological section had the lowest count of degenerative cells, while induction of epilepsy caused substantial increases in degeneration. Also, VPA treatments caused a moderate reduction in degeneration level, but LO and LX caused reduced degeneration, nearing the control group.

Regarding the thickness of the cerebellum, the control section recorded 48,948.180 mm, while we noticed the EP group by its significantly reduced thickness (38,617.459 ± 1783.434), reflecting damage. EP-VPA, EP-LO, EP-LX: Treatments led to improvement, with EP-LX surpassing the healthy animals. Incremental EP-VPA and EP-LO treatments caused significant (*p* < 0.05) improvement in the total blood vessel count, while EP-LX showed the most remarkable recovery after severe reduction in the EP group (2.000±). On the other hand, examination of the hippocampal degenerative neurons revealed that the control group had minimal degeneration (4.688 ± 0.522) in comparison to EP, which showed a sharp increase (12.604 ± 1.07), indicating significant (*p* < 0.05) hippocampal damage. Moreover, all treatments diminished the degeneration levels, with EP-LX returning closer to the control level (6.417 ± 1.4).

## 3. Discussion

The electrical imbalance in the epileptic neuron, Na^+^, K^+^, Ca^++^, and Cl^−^ ions were monitored via their quantification in serum and hippocampal tissue during the current investigation. The current findings demonstrated a reduction in Na^+^ and Ca^++^ levels in the serum of the epileptic group, accompanied by an increase in K^+^ and Cl^−^. The present study’s observations of significant deficiencies in Na^+^, K^+^, and hypocalcemia align with the conclusions drawn by Castilla-Guerra, Fernández-Moreno [22]. The hypocalcemia that researchers recorded, and electrolyte gradients across cell membranes, may contribute to significant neurological damage by influencing neuronal discharge and promoting epileptogenesis behavior, directly or indirectly. Unlike in the epileptic hippocampal tissue, Na^+^ and Ca^++^ levels showed an elevation.

In contrast, the levels of K^+^ and Cl^−^ were shown to be lowered in comparison to the control group. Similar findings were reported by Raimondo, Burman [23], who found that epileptiform activity is linked to a peak decrease in extracellular Na^+^ and a rise in intracellular Na^+^ concentration. In the hippocampus tissue, there was a drop in K^+^ and an increase in Na^+^ due to the low-active ATPase enzyme and the low-active Na^+^/K^+^ pump.

On the other hand, Abdel-Reheim, Ahmed [24] observed hypokalemia and hypernatremia in the serum of rats with epilepsy. The Na^+^-K^+^ ATPase pump is less effective when potassium contents are lowered, and corresponding neuronal hyperpolarization occurs. Inhibiting the pump results in intracellular potassium loss, neuronal depolarization, and initially spontaneous discharge [25]. In the brain, electrochemical signaling is mediated by an electrochemical gradient. The Na^+^/K^+^ pump is an active transporter that transfers ATP hydrolysis energy [26]. Ions traverse the neuronal membrane against concentration gradients and have activities related to potential action creation and maintaining other active transport mechanisms and cell volume regulation [27]. In this instance of human TLE, insufficient spatial potassium buffering by astrocytes causes glial cells and neurons to depolarize more and for more extended periods in response to activity-dependent potassium release, which may be a factor in the development of seizures [28].

When Cl^−^ accumulates intracellularly, passive redistribution is useless. Furthermore, because Cl^−^K^+^ co-transport depends on the K^+^ gradient, it loses some effectiveness during seizures. The efficiency of Cl^−^K^+^ co-transport is dependent on numerous metabolic pathways and can be influenced by hypoxia or ischemia [29]. Alterations in the extracellular ion concentrations, reductions in GABAergic function, increases in synaptic excitation, or modifications in both K^+^ and Ca^++^ currents may result from alterations in the ionic microenvironment surrounding the epileptic foci. These events all contribute to the protracted depolarization. The precise mechanism by which seizures cease remains unknown; however, they may stop when inhibitory circuits in the neuronal network become active or when the ionic environment changes, such as decreased extracellular K+, intracellular Ca++ elimination, or glutamate release [30].

Heteromeric proteins called voltage-gated ion channels produce and transmit action potentials in brain neuronal cells. They are membrane-associated proteins that carry ion currents into the cell along their concentration gradient. Genes linked to these channel genes are the most often occurring cause of hereditary epilepsies. In this study, the ionic channels protein indicated high levels of Na^+^ and Ca^++^ gates while K^+^ was decreased. SCNN1a is a voltage-gated sodium channel subunit present in the soma and dendrites of mammals. It is the most common of all the voltage-gated sodium channels, and the most frequently implicated mutation is in it [31]. Because SCN1A is needed for neurons to fire and spread action potentials [32], the genes that code for this channel are thought to play a significant role in epilepsy [33]. Voltage-gated Na+ (Nav) channels in dendritic cells also support action potentials in cortical pyramidal cells [34].

In the current study, the pilocarpine-treated rats observed an elevated level of NMDA (glutamate receptor) and a low level of CLCNC (GABA receptor). The extensive release of glutamate in susceptible areas after pilocarpine injection leads to the activation of NMDA glutamate receptors and neuronal damage through oxidation and apoptosis after Ca^++^ influx. Conversely, dendritic NMDA receptors (NMDARs) are the primary mediators of the Na+ influx. Therefore, any rises in intracellular Na^+^ occur after the high levels of neuronal activity and NMDAR activation that cause epileptic seizures. Similarly, intracellular Na^+^ accumulations lower the Na+ driving force at glutamatergic receptors, shrinking excitatory postsynaptic currents [23]. Excessive release of excitatory neurotransmitters damages cells by activating voltage-activated Ca++ channels and NMDA receptors, which let Ca^++^ into the cell. A series of metabolic alterations brought on by Ca^++^ and other ionic changes ultimately lead to cell death [35].

Moreover, the present study noticed neurotransmitter and electrolyte imbalance, recording the status of gate modification and cytokines variations. These changes were recorded with low levels of GABA and acetylcholine-esterase but high levels of glutamate, dopamine, epinephrine, and norepinephrine in the seizure-active pilocarpine epileptic rats. These events came in accordance with previous observations [36,37,38,39]. Pilocarpine administered intraperitoneally induces a significant increase in glutamate, which is vital in the maintenance and development of seizures [40]. According to a recent study, limonene, a cyclic monoterpene component of *L. dentata*, can interact with GABAA receptors and have an anxiolytic effect by boosting GABA concentration in the brain [41]. The observed antiepileptic activity of Lavandula spp. may be due to its potency to decrease the sodium current and increase the potassium current and its inhibitory effect of GABA because of its contents of Linalool, which has an inhibitory impact through gamma-aminobutyric acid (GABA-A) receptors. This effect reduces anxiety and downregulates limbic system activity [42].

The high dopamine (DA) level in the present epileptic rats was recorded by Cifelli and Grace [43] in rodent animal models of TLE. These findings also imply that DA level fluctuations modify DA’s neuromodulatory function on limbic system brain circuits. However, Werner and Coveñas [36] outlined how GABA hypoactivity can produce dopamine hyperactivity and how DA increase is caused by dopaminergic neurons being impacted by the GABAergic system’s inhibitory action through GABAA receptors. The present results are parallel to those of Li, Wang [44], who confirmed that LEO of Lavandula angustifolia reduced dopamine levels, 5-hydroxytryptamine (5-HT), and substance P. Moreover, different investigations have demonstrated that some aromatherapy constituents can inhibit 5-HT and dopamine receptors that are linked to the sensation of nausea [45].

The fact that oxidative stress is more likely to happen in the brain shows how important it is to understand how oxidative stress leads to seizures [46]. When rats were given pilocarpine, their lipid peroxidation and nitric oxide activity levels were much higher than those in the control group. However, their levels of superoxide dismutase activity were not significantly higher. They also had significantly lower levels of catalase and glutathione activity. Sudha, Rao [47] reported high amounts of MDA in the red blood cells of epileptic patients, which matched the results of the current study. Lipid peroxidation and epileptic activity have also been linked in animal models [48,49,50,51,52,53].

According to some reports, phytoflavonoids and coumarins found in natural goods can help with a few neurological problems because they are potent antioxidants. Most secondary plant chemicals are thought to stop seizures by changing how ions move through ligand- or voltage-gated ion channels. They can connect with the voltage-gated sodium ion (Na+) channels or the benzodiazepine site of the g-amino butyric acid (GABA) A receptors, among other things [54]. The current data approved that *L. dentata* could attach to NMDA glutamate receptors and stop them from sending signals. So, other researchers thought that *L. dentata* might help neurons avoid damage caused by glutamate by either blocking this glutamate-activated ionotropic receptor or calcium channels [17]. *L. dentata* oil has been demonstrated to effectively develop spatial learning deficits in Alzheimer’s patients, significantly improving performance [55] and generating neuroprotection in patients with focal cerebral ischemia [56]. One of the main ingredients in *L. dentata* oil, Linalool, has been demonstrated in experiments to reduce glutamate binding in rats [57].

The present results came parallel with others, which revealed that *L. dentata* oil distinguishably altered the desensitization and deactivation phases of AMPA subunits’ kinetics. As a result, a relationship has been established between AMPA receptors and several neurodegenerative or neuropsychiatric illnesses [58]. Consequently, the present investigation employed an electrophysiologic methodology to examine the impacts of *L. dentata* oil. Glutamate functions as a pharmaceutical target to reduce anxiety and epilepsy, and its effects are facilitated through the AMPA receptor. To discover any modulatory activity, the oil of *L. dentata* was assessed by three separate AMPA receptor characteristics: peak current, desensitization, and deactivation [58].

Examining the possible biological effects of essential oils (Eos), especially on AMPA receptors in the central nervous system, was fascinating. *L. dentata* oil has a calming effect that may be partly explained by its inhibitory effect on glutamate receptors [17]. *L. dentata* can either block the glutamate-activated ionotropic receptor or its calcium channel-blocking activity or induce neuroprotection in focal cerebral ischemia because it binds to NMDA glutamate receptors with affinity and exhibits inhibitory activity at those receptors [56] to protect against glutamate-induced toxicity. Furthermore, lavender extract’s antioxidant qualities and calcium channel blockage contribute to its neuroprotective effects [59]. Thus, suppressing glutamate release, NMDA receptors, and/or calcium channel blockage appears to mediate the antiepileptic effect of *Lavender* sp.

According to newly available data, flavonoids may help prevent neurological diseases by modulating γ-aminobutyric acid (GABA) receptors [60], mitigating mitochondrial dysfunction [61], and controlling antioxidative and anti-inflammatory mediators like superoxide dismutase (GSH), cytokines, and glutathione (GSH) [62]. The active oil and extract ingredients, Linalool, alpha-pinene, thymoquinone, and terpinene-4-ol, can bind to the GABAA receptor at the benzodiazepine site or raise GABA activity [63].

The observed low cholinergic activity after treatment with *L. dentata* may be due to cyano-carvone inhibiting lipid peroxidation and nitrite production. Therefore, there is a synchronous correlation between behavioral modification and the anticonvulsant action. Cyano-carvone consequently lengthens the latency periods before the onset of initial seizures and the establishment of status epilepticus. Cyano-carvone has also been demonstrated to counteract the behavioral alterations that are brought about by pilocarpine [64].

In addition to other primary ingredients that can account for several positive benefits, such as polyphenols, the sound effects of Lavender sp. in traditional medicine have traditionally been attributed to essential oils in the plant [65]. According to Comalada, Ballester [66], Comalada, Camuesco [67], flavonoids are polyphenols with antioxidant and immunomodulatory qualities that directly influence the anti-inflammatory effects seen in experimental models of colitis. Dietary flavonoids are recognized for their ability to trigger cytoprotective proteins and to interact with various neurotransmitter systems that may be implicated in neuroprotection, such as dopamine [68], glycine [69], GABA [70], and adenosine [71].

*L. dentata* extracts have recently demonstrated immunomodulatory qualities in vitro while downregulating various inflammatory mediators, including nitric oxide and cytokines. These actions were linked to the downregulation of proinflammatory cytokines and many inducible enzymes, including MMP-9, iNOS, and COX-2, all implicated in maintaining the inflammatory state. In the DPPH Assay, the *L. dentata* extracts exhibited comparable antiradical activity. Additionally, after receiving either *L. dentata*, the depletion of the colonic glutathione level was partially prevented in control colitic rats [72].

Moreover, NMDA receptors are antagonistic towards monoterpene elements of *L. dentata*, such as α-pinene, citronellal, and myrcene [73]. Most essential oils are made up of terpenes, which are in some way responsible for the pharmacological actions of the medicinal plant, including its antinociceptive, anti-inflammatory, and anticonvulsant properties [74]. Among the monoterpenes, alpha-pinenes are a potent acetylcholinesterase inhibitor and have a stimulating impact on GABAA receptors and boost postsynaptic GABA-dependent chloride fluxes [75]. The DAEO monoterpenes citronellal, citronellol, myrcene, and β-pinene can also block NMDA receptors and shield neurons from excessive stimulation [76]. A significant reduction in striatal dopamine (DA) and norepinephrine (NE) levels and hippocampal nitrite was observed following administration of α-pinene and an equimolar mixture of the two monoterpenes. The results of this study indicate that the potential anticonvulsant effects of β-pinene could be attributed to a reduction in the levels of the two neurotransmitters involved [77].

*L. dentata* contains borneol, which could increase GABA activity and shield neurons from harm during epileptic convulsions [78]. Previous research has demonstrated that borneol has anti-hyperalgesic effects on neuropathic and inflammatory pain by lowering mechanical hyperalgesia and enhancing GABAAR-mediated GABAergic transmission in the spinal cord [79]. Borneol can suppress GABA and glycine transmission in the substantia gelatinosa neurons of the trigeminal subnucleus caudalis. Furthermore, it directly affects postsynaptic substantia gelatinosa neurons and mediates the glycine and GABAA receptors’ activation to cause substantia gelatinosa neurons to elicit inward currents [80] repeatedly.

Furthermore, because of its hydrocarbon skeleton, which is thought to be a hydrophobic ligand that might aid in interacting with the hydrophobic active site of AChE, limonene is also reported to have anti-AChE activity [81]. Additionally, limonene can raise glutathione (GSH), catalase, superoxide dismutase (SOD), and other oxidative stress indicators such as protein carbonyls and malondialdehyde (MDA) while decreasing others [82]. Moreover, limonene was found to efficiently increase the IL-10/IL-2 ratio, which in turn increases the levels of IL-10, an anti-inflammatory cytokine synthesis inhibitory factor that inhibits the production of proinflammatory Th1 cytokines [83].

Additionally, research on Linalool, a monoterpene alcohol and one of the main ingredients of *L. dentata* oil, showed that it might prevent rats from binding glutamate [57]. Linalool has been shown to enhance GABAA function in mammalian electrophysiology studies [84]. Furthermore, compounds derived from Linalool and its metabolites, including Linalool oxide, linalyl acetate, 8-oxo linalyl acetate, 8-carboxy linalyl acetate, and 8-oxo Linalool, have been observed to influence GABAA activity and have anticonvulsive properties [84,85]. Furthermore, lavender extracts’ antioxidant qualities and calcium channel blockage may contribute to their documented neuroprotective benefits [59]. Thus, suppressing glutamate release, NMDA receptors, and/or calcium channel blockage appears to mediate the antiepileptic effect of Lavender sp. One of the essential oils of lavender, Linalool, has been shown in human embryonic kidney cells to inhibit T-type calcium channels [86]. As a result, it may attenuate cellular excitability by lowering intracellular calcium and providing additional protection against calcium toxicity during seizure episodes.

Epilepsy may be a progressive disease by positing that it affects multiple brain regions simultaneously but to varying degrees [87]. In addition to improving memory impairment, valproate (VPA) and phenytoin (PHT) have been proven to raise synapse-related gene mRNA expression, and protein levels [88] also showed that epilepsy increases the risk of generating dementia and memory impairment. Hence, LEO can potentially produce an antiemetic impact by suppressing receptors associated with 5-HT and obstructing the subsequent Ca2+/CaMKII/ERK1/2 pathway of the cAMP signaling pathway [44].

The recorded effector molecules, such as TNF-α, TGF-β, IL-1, IL-6, IL-8, and oxygen-free radicals like reactive oxygen species, are continuously secreted by microglia that are activated by recurrent seizures. These molecules can harm other neurons, glial cells, and the blood–brain barrier, causing local or extensive damage to the central nervous system, leading to epilepsy, and inducing seizures. The current study’s high levels of inflammatory cytokines caused by epileptogenic pilocarpine are in line with the results of earlier investigations [89]. In the epileptic group, the current study identified an increase in IL-6, IL-17, TNF-α, TGF-β, FOXP3, and CTLA-4 and a reduction in IL-10. Previous research has established the correlation between local blood–brain barrier breakdown and TGF-β signaling in glia and local inflammation of the cerebral cortex microenvironment [90]. Other studies have demonstrated this correlation by activating a TGF-β receptor-mediated signaling cascade in glia, which subsequently induces local inflammation. The current study suggested the inhibitory activity of the monoterpene p-cymene, which reduced TBARS formation, indicating a protective antioxidant effect. It is imperative to acknowledge that p-cymene exhibits two potential antioxidant reactions: the generation of lipid radicals and the synthesis of nitrite radicals. This is attributed to the compound possessing two locations containing benzylic hydrogens [91]. Consistent with earlier studies, p-cymene is highly effective in preventing the production of reactive species from nitrogen, which is thought to have a neuroprotective effect.

In addition, *L. dentata’s* active ingredient, borneol, can raise GSH, SOD, and CAT levels simultaneously, demonstrating a protective effect against oxidative stress brought on by PTZ [92]. Furthermore, by lowering the degree of DNA damage, borneol treatment decreased the genotoxicity of H_2_O_2_ in both testicular cells and hepatocytes in vivo [93]. Furthermore, borneol reduces the production of reactive oxygen species (ROS) in the neurons in the rats’ cerebral cortex, promoting neuroprotection by decreasing the expression and activation of inducible nitric oxide synthase (iNOS) and preventing neuronal death [94]. The borneol content of *L. dentata* can inhibit oxidative stress and neuroinflammation in astrocytes by decreasing the level of GFAP in the brain, which decreases LPO and raises the levels of SOD, GSH, and CAT [95]. As a terpene derivative, borneol can also improve the ultrastructure of neurons by lowering the apoptotic index and intracellular calcium levels in both the brain and the hippocampus. Borneol can lower the Bax/Bcl-2 ratio and TNF expression in the hypothalamus to reduce [Ca2+] overload and protect neurons in different paths from dying [96].

Another *L. dentata’s* thymol—a natural phenolic monoterpenoid, from p-Cymene, the same isomer as carvacrol—has been proposed to be a neuroprotective component in cortical rat neurons against oxidative stress caused by H_2_O_2_ [97]. In vitro, thymol has been demonstrated to exhibit antioxidant capabilities [98], and a high capacity for hydroxyl radical elimination [99]. Thymol can raise the levels of non-enzymatic antioxidants such as vitamins C and E, as well as antioxidant enzymes like SOD, GPx, CAT, and GST [100]. Thymol treatment, which increases GSH levels, lowers lipid peroxidation, and lowers blood MDA levels, can dramatically lessen the neurotoxic effects of amyloid on rat hippocampal neurons [101].

Recently, it was reported that some flavonoids like quercetin could lessen seizures and neuronal death via lowering astrocytic activation, according to a study on the anti-inflammatory effect of flavonoids in experimentally generated epileptic convulsions. This suggests flavonoids provide neuroprotection during seizures by modulating inflammatory responses and their antioxidant effects [102]. Moreover, *Lippia multiflora’s* plant was recorded with dual antiepileptic and anti-inflammatory qualities and linked to a significant decrease in seizure frequency and intensity. This implies that oxidative stress and inflammation, two critical elements in the pathogenesis of epilepsy, can be successfully addressed by herbal extracts [103].

Moreover, Azimi, Rahmati [104] have demonstrated that the administration of lavender has the effect of delaying the emergence of status epilepticus, reducing its length, and decreasing the associated death rate. The cells exposed to *Lavandula dentata* extract exhibited elevated levels of glutathione, a crucial element in bolstering cellular defenses against oxidative stress. The recent literature confirms that the antioxidant compounds in *L. dentata* exhibited vigorous antioxidant activity with an IC50 value of 16.41 ± 0.11 µg/mL in the DPPH radical scavenging assay [105]. Therefore, *L. dentata* can help mitigate the effects of reduced GPx activity by enhancing overall antioxidant defenses in the body. Also, these chemical contents of *L. dentata* act as additional sources of antioxidants and, therefore, can potentially compensate for the decreased levels and functions of GPx function, i.e., reducing oxidative stress levels. Another study highlights that flavonoids can enhance antioxidant defenses by modifying enzymes like glutathione peroxidase and superoxide dismutase, suppress inflammatory mediators, and disrupt inflammatory signaling pathways (e.g., NF-κB, MAPK). Both oxidative stress and neuroinflammation are essential factors in the pathophysiology of epilepsy, and this combined action may help reduce them [106]. In addition, the flavonoids of Ganoderma lucidum have antiepileptic effects through anti-inflammatory processes, such as blocking the production of NF-κB in the brain. Possible antioxidant effects accompany this decrease in neuroinflammation, indicating that the extract may have dual protective benefits against seizures by reducing inflammation and oxidative stress [107].

Furthermore, carvacrol, an active ingredient of the monoterpenoid phenol, has been shown to decrease the production of IFN-g, IL-6, and IL-17 [108], partly by regulating auto-reactive Th1 and Th17 cells. Furthermore, carvacrol inhibits severe leukocyte influx into the CNS by decreasing inflammatory cytokines implicated in the local production of chemokines and adhesion molecules [109].

Vieira, de Oliveira [110] predicted that the epileptic individuals’ cells would exhibit an activated profile compared to controls due to earlier research showing elevated levels of circulating pro-inflammatory cytokines and/or mediators, indicating chronic low-grade peripheral inflammation in TLE. In instances of epileptogenesis, elevated inflammatory cytokine levels can break down the blood–brain barrier, allowing diverse immune components to interact with one another freely. The abnormal neuronal hyper-excitability resulting from these inflammatory mediators’ production will increase the permeability of glutamatergic neurons to calcium ions [111]. The authors examined two waves of inflammatory mediators and cytokines from neurons or glial cells, which can intensify downstream cascades of inflammation and cause neuronal hyper-excitation during epileptogenesis.

Adult brain cells express a variety of pro- and anti-inflammatory cytokines, including TNF-a, IL-1b, IL-6, IL-8, IL-10, IL-12, and IL-15, and several chemokines, including CCL2, CCL3, and CCL4. These have been reviewed by Lee, Nagai [112], and Wang, Wang [113]. While many studies—Sinha, Patil [114] Mao, Ding [115]; Pernhorst, Herms [116]; Sonmez, Serin [117]—have identified a correlation between IL-4, IL-8, and IL-17 levels and seizure frequency and severity, other studies have not found an association between these parameters. Finally, Wang, Wang [113] found that in three distinct types of epilepsy, namely temporal lobe epilepsy (TLE), extra-temporal lobe epilepsy (XLE), and idiopathic generalized epilepsy (IGE), only four independent cytokine biomarkers (IL-6, IFN-γ, IL-17-a, and IFN-λ3) are correlated with severe seizures.

Moreover, the present data assumes that there is a correlation between oxidative stress markers and IL-17 in epilepsy, highlighting the potential of therapeutic strategies of using *L. dentata* oils or extracts targeting inflammation and oxidative damage. Postulated anti-inflammatory therapies of the current herbal treatment aimed at reducing oxidative stress may contribute to lowering atherosclerosis, while antioxidants targeting IL-17 may reduce oxidative stress and can reduce the frequency and severity of seizures [118]. Recently, many studies showed that high levels of IL-17 can increase oxidative stress by activating inflammatory pathways that increase ROS production [119]; for example, IL-17 can stimulate pro-inflammatory cytokines such as TNF-α and IL-6. This bidirectional relationship with IL-17 increases brain tissue’s recorded oxidative stress response. Also, IL-17 promotes oxidative stress through inflammatory mechanisms, and ROS can also increase IL-17 expression, indicating a feedback loop that exacerbates neuroinflammation and oxidative damage during epileptic events [120].

The recorded attenuation of the inflammatory cytokines may be due to the presence of borneol, one of the terpene derivatives in the extract or oil of *L. dentata*. By inhibiting p-p65 and p38 signal transduction, borneol protects neurons and microglia from inflammatory activity by reducing LPS-induced inflammation, decreasing TNF, IL-1, and IL-6 levels in the brain, and increasing IL-10 [121]. Regarding the assessment of intracellular cytokine production, the present study identified a rise in IL-6, TNF-a, and IL-17. This finding aligns with prior research that documented increased cytokine concentrations in lymphocytes and monocytes, cerebrospinal fluid (CSF), and serum/plasma during the interictal period, specifically following seizures, in patients with TLE and other epileptic syndromes [122]. Furthermore, IL-10’s neuroprotection, cell survival, and anti-inflammatory properties are linked to its ability to protect the central nervous system from various proapoptotic factors created during immune response activation [123]. The effects of IL-10 on cell death include blocking initiator and effector molecules [124], preventing TNF-α-induced cell death in astrocytes [125], reducing cerebellar neuron death through NMDA receptor-induced excitotoxicity, inhibiting mitochondrial-induced cell death in excitotoxicity [126,127], inhibiting mitochondrial-induced cell death in microglia [128], and, lastly, blocking the cascade of apoptosis [129]. Elevated concentrations of interleukin-6 (IL-6), interferon-gamma (IFN-γ), and interleukin-17A (IL-17A) have been observed in the plasma during the interictal phase of epilepsy, whereas IL-1β, TNF-α, and IL-10 did not rise in comparison to a healthy group [130].

Conversely, Foxp3 expression must remain constant in mature Treg cells for them to continue acting as suppressors [131]. Foxp3 in glial cells has been demonstrated to serve a protective role in the pathophysiology of epilepsy [132]. Foxp3 plays a neuroprotective effect in these cells by downregulating TLR4 signaling, which involves NMDA receptor deactivation to lessen neuronal death. Treg formation and function regulators like Foxp3 can impact the ratio of anti-inflammatory Tregs to pro-inflammatory Th17 cells [133].

The observed neuroprotective action of *L. dentata* to restore the histopathological alterations—recently, carnosol content of Lavander spp.—could prevent cell death in dopamine neuron cells and increase the production of tyrosine hydroxylase. This effect is achieved by activating the Raf-MEK-ERK1/2 pathway [134]. The more significant cognitive functions, such as memory creation and consolidation, are thought to be primarily mediated by the hippocampus [135]. Hippocampal cell loss is shown histologically in the CA1, CA3, and dentata hilus, following a pattern like that of patients with mesial temporal sclerosis and persistent temporal lobe epilepsy [136]. According to Bui, Nguyen [137], the present investigation revealed that in rats with chronic epilepsy, there was a notable reduction in the activation of mossy cells. These cells are glutamatergic populations located in the hilum of the hippocampal dentata gyrus (DG). These drops alone hindered spatial contextual processing and resulted in memory impairments. Also, the hippocampus is a part of the brain that can cause seizures and is easily damaged by them. Therefore, hippocampal sclerosis frequently manifests as a neuropathological characteristic of epilepsy. The occurrence of this phenomenon is observed in around 30 to 45% of all epileptic syndromes, with a higher prevalence of 56%, specifically in the case of MTLE, as reported by Thom in 2014. Hippocampal sclerosis is the prevailing pathological condition associated with drug-resistant epilepsy in the elderly population [138].

Collectively, image analysis and enumeration of different inflamed areas showed that the EP group had significant damage across all metrics, indicating the adverse effects of the experimental condition. In contrast, EP-VPA had moderate recovery across metrics, suggesting the treatment is partially effective, and *L. dentata* extract showed more pronounced recovery, with EP_LX (higher dose) consistently outperforming *L. dentata’s* oil. Therefore, the experimental induction of epilepsy severely impacts brain health, causing reduced neuron and glial cell counts, increased degeneration, and structural damage. At the same time, *L. dentata’s* oil treatment improves outcomes, with treatment with *L. dentata’s* extract being the most effective, nearing or exceeding control levels in some metrics.

## 4. Materials and Methods

### 4.1. Animals

Forty mature male Wister rats, weighing 150–180 g, were obtained from the animal laboratory at the National Research Institute in Dokki, Giza, Egypt. The subjects were housed in separate metabolic cages within a controlled environment, maintaining a temperature of 23 ± 1 °C and a humidity level of 55%. The lighting conditions followed a 12 h light/dark cycle. A wide range of drinks and food was readily accessible. The experimental protocols adhered to the guidelines for the care and utilization of laboratory animals and were adequately reviewed by the animal research ethics committee of Beni-Suef University, with the assigned approval number BSU/FS/2021/021/138.

### 4.2. Pharmaceuticals and Chemicals

The pharmaceuticals and chemicals utilized in this study included pilocarpine hydrochloride (99%) (PILO), which was procured from MP Biomedicals located in Solon, OH, USA (SKU:02151892-CF). Valium and methylscopolamine were procured from Nanjing Chemical Reagent Co., Ltd., located in Nanjing, China. The valproic acid, specifically the brand Depakine Chrono^®^500 mg, was purchased from Sanofi Aventis CO., Paris, France a pharmaceutical company. The valproic acid falls under the Anatomical Therapeutic Chemical (ATC) classification code N03AG01.

### 4.3. Lavandula Dentata Collection, Extraction, and Characterization

Aerial parts of *Lavandula dentata* L. were collected from the Taif region in Saudi Arabia in May 2018 when the plant was still growing. A voucher specimen was deposited in the herbarium. In contrast, it was identified, authenticated, and described with the aid of the Flora & Phyto-Taxonomy Research, Horticultural Research Institute, Agricultural Research Centre 12618, Dokki, Cairo, Egypt. Briefly, extraction and characterization of *Lavandula dentata* L.: About 250 whole dried plant material was coarsely milled and extracted with 70% ethanol via ultrasonic assistant extraction bath at room temperature till exhaustion (3 times). The pooled ethanol extracts were evaporated under a vacuum at 45 °C till evaporation of the organic solvent, and part of the remaining aqueous extract was freeze-dried to produce 47 g of greenish residue. Chemical component identification was accomplished by comparing mass spectra with genuine standards from the NIST/EPA/MSDC Mass Spectral Database and retention indices concerning alkanes (C8–C20). Peak area normalization was used to generate quantitative data without the need for correction factors [139,140,141]).

### 4.4. Induction of Epilepsy (In Vivo)

Seizure induction was applied according to Turski, Ikonomidou [142], and Abdel-Reheim [143]. Briefly, before receiving 300 mg/kg of pilocarpine hydrochloride (PILO), each study rat received a dose of 1 mg/kg of methylscopolamine for 30 min. Rats’ behavior was observed for 120 min to determine whether or not specific criteria were met for seizure activity. An epileptic’s ability to exhibit symptoms of convulsions, tremors, salivation, and drowsiness was one of the conditions for becoming a good model.

### 4.5. Animal Grouping

Forty grown male Wister rats weighing between 150 and 180 g were used in this study. They were split into five groups of eight rats each. The groups were as follows: (1) The control group (C) was fed a standard meal and had unlimited access to sterile water. At the same time as the treatment groups, they were orally administered PBS (pH 7.4; 0.2 mL/rat) via an intragastric tube. (2) Epileptic group (EP) was given 300 mg/kg of PILO injection, as previously described [143]. (3) The group of epileptic rats treated with valproic acid (EP-VPA) was administered by the drug via intragastric intubation. Following this, they received an injection of 300 mg/kg of PILO, as described in earlier studies (Raza, Dhariwal [144] and Ortinski and Meador [145]). Additionally, the rats were orally fed 500 mg/kg of VPA diluted in PBS (pH 7.4; 0.2 mL/rat). The VPA sessions were conducted twice a week for a consecutive duration of four weeks, agreeing with the scheduling pattern observed in the other groups [146]. (4) The epileptic group receiving *L. dentata* pure oil treatment (EP-LO): Following the previously mentioned injection of 300 mg/kg of PILO, the rats were given an oral diet containing 100 μg of *L. dentata* oil per kilogram of body weight, suspended in PBSS (pH 7.4; 0.2 mL/rat). Using intragastric intubation, the oil feeding was carried out every day for four weeks at intervals consistent with the other groups. (5) The epileptic group receiving *L. dentata* pure oil treatment (EP-LX): Following the previously mentioned injection of 300 mg/kg of PILO, rats were fed a liquid containing 300 mg/kg of ethanol extract of *L. dentata* mixed with PBSS (pH 7.4; 0.2 mL/rat). The extract feeding was carried out every day for four weeks at intervals consistent with the other groups by inserting a tube into the stomach.

### 4.6. Blood and Tissue Sampling and Biochemical Measurements

Neurotransmitters and electrolytes were assessed in the hippocampal area of the brain, which was cut open and put on plates filled with ice. A Potter–Elvehjem homogenizer (Braun, Melsungen, Germany) was used to mix pieces of hippocampal tissue in cold PBS (pH 7.4) with eight up and down strokes of a loose-fitting Teflon pestle with a weight of 1000 g. After filtering, the mixture was put in a Beckman TJ-6 centrifuge (Beckman Instruments; Munich, Germany) and spun at 600× *g* for 10 min at 4 °C. At −80 °C, the clear supernatant was kept until many papers were examined. The amount of total protein in the supernatant (/gm tissue) was determined [147]. Portions of brain tissue slices were stored at −80 °C in sterile Eppendorf tubes until they were needed for RT-PCR analysis and RNA extraction. The procedure for making neurotransmitters followed the purchased manufacturer’s instructions. Levels of L-DOPA (µg/mL), epinephrine (ng/mL), norepinephrine (pg/mL), glutamate (µg/mL), and gamma-aminobutyric acid (GABA, pg/mL) were measured using assay kits from MyBioSource Co. Vancouver, British Columbia: MBS 9357024, MBS 031232, MBS 269993, MBS 047402, and MBS 269152 were the kit catalog numbers. Acetylcholinesterase (U/L) activity was measured by following the instructions provided with the QuantiChromTM Assay Kit (Cat. No. DACE-100). The electrolyte content of both potassium (K+) and sodium (Na+) (mMol/L) were measured in either brain homogenate tissue or blood, according to the MyBioSource handbook. In addition, QuantiChromTM assay’s quantitative colorimetric instruction booklet was also utilized to determine the amounts of calcium (Ca^++^) and chloride (Cl^−^) in brain homogenate tissue or blood.

### 4.7. Assessment of Oxidative Stress

Prooxidant and antioxidant activities were assessed in brain homogenate tissues by the production guide purchased from MyBioSource Co. and included with the test kits. Malondialdehyde (MDA) was discovered in the hippocampal region to indicate lipid peroxidation. Thiobarbituric acid reactive substances (TBARSs) in tissue samples were quantified as malondialdehyde (MDA) using spectrophotometry [148]. Nitric oxide (NO) accelerates the conversion of oxyhemoglobin to methemoglobin, which can be monitored via spectrophotometric measurement [149]. The GPx activity was measured using T-butyl-HPx as a substrate [150]. The quantity of nitro blue tetrazolium (NitroBT) that was decreased with NADH by phenazine methosulfate (PMS) under aerobic circumstances was used to determine the amount of superoxide dismutase (SOD) activity. Adding or being in the presence of superoxide dismutase stopped this reaction [151]. One way to measure catalase (CAT) activity is to watch the loss of H_2_O_2_ using spectrophotometry [49].

### 4.8. Isolation of RNA and Quantitative Reverse Transcription Polymerase Chain Reaction (qRT-PCR)

The process of isolating RNA and performing PCR was done using real-time quantification. The SYBR Green I analysis was conducted using an implemented BioSystem with software version 3.1 (StepOneTM, USA). The qPCR test designates the target primer sequence at a temperature of 60 °C for one minute, which agrees with the annealing temperature. The denaturation temperature was set at 95 °C for one minute, while the extension temperature was maintained at 72 °C for the same duration. This was done 40 times. The comparative threshold (CT) method was used to measure gene expression compared to other genes [152,153]. The numbers were worked out after comparing the changes in mRNA transcript level to GAPDH. Each experiment was done three times, each time on its own. PCR (RT–PCR) with the following rat-specific primers: Spectrophotometry is used to track the SCN1A (Na+ channel) of H_2_O_2_ [36]: F; 5-TCATGGCACAGTTCCTGTATC-3; R; 5-GCAGTAGGCAATTAGCAGCAA-3; Kcnj2 (K+ channel): F; 5-GCAAACTCTGCTTGATGTGG-3; R; 5-TCATACAAAGGGCTGTCTTCG-3; CACNA1S (Ca2+ channel): F; 5-GACATAATTCCCGCTGCCTG-3; R; 5-GTTTCCATTCTTCACCCGCC-3; CLCN2 (GABA-receptor): F; 5-CACTGGATAACAACGCCCA-3; R; 5-GCAGGGAATGTAGGTCTGG-3; NMDA (Glutamate Receptor): F; 5-ACTCCACACTGCCCATGAAC-3; R; 5-TTGTTCCCCAAGAGTTTGCTT-3; FOXP3: F; 5-TCATCCGCTGGGCCATCCTG-3; R; 5-GTGGAAACCTCACTTCTTGGTC-3; CTLA4: F; 5-GGACGCAGATTTATGTCATTGATC-3 and R; 5-CCAAGCTAACTGCGACAAGGA-3; and GAPDH: F; 5-GTGAAGGTCGGAGTCAACG-3; R; 5-CAATGCCAGCCCCAGCG-3.

### 4.9. Histopathological Investigation

Brain tissue samples were taken and put in a 10% neutral buffered formalin solution (Sigma, St. Louis, MO, USA) to keep them still for 24 h [154]. To sum up, tap water was used to wash the tissues, and then a run of diluted ethyl alcohol was used to dry them out. Animal parts were cleaned with xylene and then put in a hot oven at 56 °C for 24 h to bury in paraffin. To cut the tissue into five μm slices with a microtome (Leica Microsystems, San Francisco, CA, USA), paraffin wax blocks were first made. The tissue sections were placed onto glass slides and subjected to deparaffinization using xylene, followed by hydration using a sequential sequence of alcohols with concentrations of 100%, 95%, 90%, 80%, and 70%. Subsequently, the specimens were immersed in water and subjected to staining using hematoxylin and eosin (H&E) obtained from Sigma. The slides were subsequently examined using a conventional light microscope manufactured by Olympus Optical Corporation in Tokyo, Japan. The scoring system utilized in this study assessed brain alterations, specifically nuclear pyknosis and degeneration in neurons, localized gliosis, and encephalomalacia, on a scale ranging from 0 to 3 (0 meant the brain was normal, 1 meant mild damage, 2 meant moderate damage, and 3 meant severe damage) [155].

All possible quantifications for the image analysis were done for the studied layers, e.g., the cerebral cortex, cerebellum, and hippocampus regions. All cells count measurements were performed using the program ImageJ (https://imagej.nih.gov/ij/), and the cell density, number of blood vessels, and layer thickness were examined in all H&E images (six fields/rat/group) [156]. The number of cells per unit area (cells/µm^2^) was also determined, while the fluctuations were measured and tracked. A thorough examination of the differences in cell density between the various rat groups was made possible by this methodical approach, which enabled us to acquire consistent data for the groups.

### 4.10. Immunohistological Tissue Staining

The primary and secondary antibodies were purchased from Abcam (Cambridge, MA, USA), which included anti-FoxP3 (Cat. #: AB75763) as the primary antibody and Goat Anti-Rabbit IgG H&L (DyLight 488 pre-adsorbed, AB96899) as the secondary antibodies. Immunohistochemistry (Formalin/PFA-fixed paraffin-embedded sections) was performed on rat brain tissue staining FOXP3 using antibody CAT # ab75763 with final conc. 1/100, DAB staining (in brown) and hematoxylin QS as a counterstain (in blue). ImageJ software was used to analyze the histoarchitecture of the FOXP3-positive cells. Six animals were used in each experimental group to gather quantitative data; each animal had 3 to 4 high power fields to be examined, and the values were averaged to represent the animal.

### 4.11. Statistical Analysis

The Shapiro–Wilk test was utilized to verify if the current data were expected. Means and standard deviation were employed because the data were regularly distributed. If there was no homogeneity of variance, the Welch Test, a modified ANOVA, was used. The Tukey–Kramer method was employed for doing post hoc analysis of the data, to compare various groups. The means of homogenous subsets’ groups are presented, with each value represented as Mean ± Std and a sample size of n = 8 animals per group. A notable statistical contrast at a significance level of *p* < 0.05, relating to the same variable and lacking a shared superscript symbol or symbols, was indicated. The data analysis was conducted using the Statistical Package for Social Science (SPSS) version 20 software program (IBM Corp., 2011).

## 5. Conclusions

*Lavandula dentata* enhances the activity of antioxidant enzymes such as GSH, SOD, and CAT, protecting against oxidative stress generated by PTZ. *L. dentata* could reduce inflammatory cytokines’ effects and protect neurons and microglia against inflammation. Currently, no research specifically investigates the impact of *L. dentata* oil and ethanol extracts on FoxP3+ regulatory T cells (Treg) in a rat model of epilepsy. However, the available data suggests that *L. dentata’s* oil and extract may benefit by restoring the balance between pro-inflammatory Th17 cells and anti-inflammatory Treg cells, including those expressing Foxp3. Therefore, based on the current study’s findings, the practical applications of *Lavandula dentata* extracts include their integration into therapeutic regimens, neuroprotective supplement use, and the development of phytopharmaceuticals. These extracts could enhance seizure control and mitigate cognitive side effects if used alongside standard antiepileptic drugs (AEDs) to enhance seizure control while mitigating cognitive side effects often associated with AEDs. Moreover, *Lavandula dentata* supplementation could also be used as a natural way to support brain health in individuals with seizures or neurodegenerative conditions.

## Figures and Tables

**Table 1:** Changes in electrolytes in sera (mmol/L) and hippocampal homogenate tissues (mmol/L Tissue) of different groups: control (C), epileptic (EP), VPA-treated (EP-VPA), *L. dentata* oil-treated (EP-LO) and *L. dentata* extract-treated (EP-LX). The data were analyzed using the Tukey–Kramer method for post hoc analysis to compare various groups with each other. Subset for alpha = 0.05 and the means for groups in homogeneous subsets are displayed. All values were represented as Mean ± Std and n = 8 animals/group. Means within the same parameter and not sharing a common superscript symbol(s) differ significantly at *p* < 0.05.

Group	Na^+^		K^+^		Ca^++^		Cl^−^	
	Serum (mmol/L)	Tissues (mmol/L Homogenate Tissue)	Serum (mmol/L)	Tissues (mmol/L Homogenate Tissue)	Serum (mmol/L)	Tissues (mmol/L Homogenate Tissue)	Serum (mmol/L)	Tissues (mmol/L Homogenate Tissue)
C	139.75 ± 0.51 ^c^	22.69 ± 0.71 ^a^	4.86 ± 0.51 ^a^	90.77 ± 10.81 ^b^	1.03 ± 0.17 ^d^	0.30 ± 0.06 ^ab^	105.06 ± 3.65 ^a^	94.17 ± 3.52 ^b^
EP	128.89 ± 0.46 ^a^	29.07 ± 0.56 ^b^	7.13 ± 0.28 ^c^	65.63 ± 6.62 ^a^	0.36 ± 0.08 ^a^	0.60 ± 0.02 ^d^	115.57 ± 2.77 ^b^	73.08 ± 6.45 ^a^
EP-VPA	137.55 ± 1.93 ^bc^	20.43 ± 1.07 ^a^	5.70 ± 0.15 ^b^	86.27 ± 4.89 ^b^	0.81 ± 0.05 ^bc^	0.29 ± 0.02 ^a^	103.31 ± 2.42 ^a^	91.29 ± 6.15 ^b^
EP-LO	138.44 ± 1.12 ^bc^	21.70 ± 0.33 ^a^	4.35 ± 0.14 ^a^	92.78 ± 5.15 ^b^	0.76 ± 0.12 ^b^	0.39 ± 0.03 ^c^	103.32 ± 3.01 ^a^	95.40 ± 2.50 ^b^
EP-LX	136.59 ± 1.17 ^c^	26.51 ± 2.47 ^bc^	4.65 ± 0.54 ^a^	80.69 ± 3.79 ^b^	0.99 ± 0.08 ^cd^	0.36 ± 0.02 ^bc^	106.48 ± 3.93 ^a^	105.81 ± 1.57 ^c^
F value	40.304	24.021	28.086	7.879	17.957	41.937	7.620	21.096
*p*<	0.000	0.000	0.000	0.004	0.000	0.000	0.004	0.000

**Table 2:** Changes in mRNA gene expression (relative to control) for some gate receptors of electrolytes and neurotransmitters in brain tissues of different groups: control (C), epileptic (EP), VPA-treated (EP-VPA), *L. dentata* oil-treated (EP-LO) and *L. dentata* extract-treated (EP-LX). The data were analyzed using the Tukey–Kramer method for post hoc analysis to compare various groups with each other. Subset for alpha = 0.05 and the means for groups in homogeneous subsets are displayed. All values are represented as Mean ± Std and n = 8 animals/group. Means within the same parameter and not sharing a common superscript symbol(s) differ significantly at *p* < 0.05.

Group	SCN1A (Na^+^)	KCNJ2 (K^+^)	CACNA1S Ca^++^; (Cav1.1)	CLCNC (GABA Receptor)	NMDA (Glutamate Receptor)
C	1.01 ± 0.01 ^b^	1.01 ± 0.01 ^c^	1.01 ± 0.01 ^a^	1.03 ± 0.02 ^d^	1.01 ± 0.01 ^a^
EP	1.44 ± 0.01 ^c^	0.36 ± 0.05 ^a^	7.07 ± 0.40 ^c^	0.14 ± 0.03 ^a^	7.71 ± 1.63 ^c^
EP-VPA	0.79 ± 0.03 ^a^	0.62 ± 0.05 ^b^	3.17 ± 0.35 ^b^	0.62 ± 0.03 ^b^	3.73 ± 0.20 ^b^
EP-LO	0.81 ± 0.05 ^a^	0.65 ± 0.10 ^b^	2.87 ± 0.33 ^b^	0.77 ± 0.08 ^c^	3.22 ± 0.58 ^b^
EP-LX	0.78 ± 0.05 ^a^	0.58 ± 0.08 ^b^	2.54 ± 0.60 ^b^	0.56 ± 0.06 ^b^	3.64 ± 0.34 ^b^
F value	214.089	41.896	101.207	128.050	28.040
*p*<	0.000	0.000	0.000	0.000	0.000

**Table 3:** Estimation of some neurotransmitters in brain tissues homogenate by ELISA of different groups: control (C), epileptic (EP), VPA-treated (EP-VPA^®^), *L. dentata* oil-treated (EP-LO), and *L. dentata* extract-treated (EP-LX). The data were analyzed using the Tukey–Kramer method for post hoc analysis to compare various groups with each other. Subset for alpha = 0.05 and the means for groups in homogeneous subsets are displayed. All values were represented as Mean ± Std and n = 8 animals/group. Means within the same parameter and not sharing a common superscript symbol(s) differ significantly at *p* < 0.05.

Group	DOPA (Ug/ Protein Tissue	Epinepherine (EP) (pg/mg Protein Tissue)	Norepinephrine (NE) (pg/mg Protein Tissue)	GABA (pg/mg Protein Tissue)	Glutamate (ng/mg Protein Tissue)	Ach-Esterase (U/mg Protein Tissue)
C	15.23 ± 1.753 ^a^	78.63 ± 2.143 ^a^	108.67 ± 3.299 ^a^	57.23 ± 0.689 ^d^	3.63 ± 0.696 ^a^	95.43 ± 3.552 ^c^
EP	36.40 ± 2.352 ^b^	134.67 ± 5.860 ^c^	213.10 ± 8.365 ^d^	19.13 ± 1.203 ^a^	24.10 ± 2.065 ^d^	38.43 ± 3.896 ^a^
EP-VPA	16.97 ± 1.503 ^a^	91.67 ± 2.410 ^b^	147.30 ± 4.613 ^bc^	43.50 ± 2.376 ^bc^	7.50 ± 1.273 ^ab^	73.10 ± 4.351 ^b^
EP-LO	17.30 ± 1.007 ^a^	92.83 ± 1.849 ^b^	133.03 ± 1.590 ^b^	49.03 ± 0.884 ^c^	10.83 ± 0.406 ^c^	70.87 ± 4.532 ^b^
EP-LX	15.30 ± 1.012 ^a^	93.83 ± 2.167 ^b^	152.70 ± 4.293 ^bc^	42.80 ± 2.892 ^b^	6.99 ± 1.011 ^ab^	89.57 ± 4.535 ^c^
F value	31.977	42.643	60.807	60.347	41.944	28.122
*p*<	0.000	0.000	0.000	0.000	0.000	0.000

**Table 4:** Image analysis quantification for histopathological alterations in the cerebral cortex, which revealed variations in all H&E photographs analyzed (six fields/rat/group), using software ImageJ, of different groups: control (C), epileptic (EP), VPA-treated (EP-VPA^®^), *L. dentata* oil-treated (EP-LO), and *L. dentata* extract-treated (EP-LX). The data were analyzed using the Tukey–Kramer method for post hoc analysis to compare various groups with each other. Subset for alpha = 0.05 and the means for groups in homogeneous subsets are displayed. All values are represented as Mean ± Std and n = 8 animals/group. Means within the same parameter and not sharing a common superscript symbol(s) differ significantly at *p* < 0.05.

Groups	Average Cerebral Neurons (cells/mm^2^)	Degenerative Neurons (cells/mm^2^)	Average Glial (cells/m^2^)	Cerebellum Thickness (mm)	Number of Blood Vessels	Hippocampus Degenerative Neurons (cells/mm^2^)
Control	25.500 ± 2.739 ^c^	10.313 ± 1.559 ^a^	42.333 ± 2.582 ^c^	48,948.180 ± 1997.897 ^c^	7.167 ± 1.722 ^c^	4.688 ± 0.552 ^a^
EP	13.333 ± 2.251 ^a^	19.254 ± 2.451 ^c^	29.500 ± 4.764 ^a^	38,617.459 ± 1783.434 ^a^	2.000 ± 1.095 ^a^	12.604 ± 1.074 ^b^
EP-VPA	20.333 ± 1.633 ^b^	14.844 ± 3.575 ^b^	40.167 ± 1.941 ^bc^	44,809.778 ± 1196.650 ^b^	3.333 ± 1.366 ^ab^	11.083 ± 1.139 ^b^
EP-LO	21.167 ± 2.137 ^b^	10.067 ± 0.467 ^a^	36.167 ± 2.229 ^b^	49,689.334 ± 2101.564 ^c^	4.500 ± 1.049 ^b^	6.417 ± 1.400 ^a^
EP-LX	22.167 ± 3.189 ^bc^	10.067 ± 0.467 ^a^	36.333 ± 2.805 ^b^	49,856.001 ± 3211.469 ^c^	5.000 ± 1.414 ^b^	6.417 ± 1.400 ^a^
F value	19.925	23.175	15.633	29.683	12.254	52.127
*p* <	0.0000	0.0000	0.0000	0.0000	0.0000	0.0000

## Data Availability

The datasets (either Tables and Figures) generated and analyzed during the current study are available from the corresponding author upon reasonable request. All data generated or analyzed during this study are included in this published articlewill be available from the corresponding author on reasonable request.

## References

[1] Vezzani A., Fujinami R.S., White H.S., Preux P.-M., Blümcke I., Sander J.W., Löscher W. (2016). Infections, inflammation and epilepsy. Acta Neuropathol..

[2] Pires G., Leitner D., Drummond E., Kanshin E., Nayak S., Askenazi M., Faustin A., Friedman D., Debure L., Ueberheide B. (2021). Proteomic differences in the hippocampus and cortex of epilepsy brain tissue. Brain Commun..

[3] Stafstrom C.E., Carmant L. (2015). Seizures and epilepsy: An overview for neuroscientists. Cold Spring Harb. Perspect. Med..

[4] Ahmad M. (2013). Protective effects of curcumin against lithium–pilocarpine induced status epilepticus, cognitive dysfunction and oxidative stress in young rats. Saudi J. Biol. Sci..

[5] Freitas R.M., Vasconcelos S.M., Souza F.C., Viana G.S., Fonteles M.M. (2005). Oxidative stress in the hippocampus after pilocarpine-induced status epilepticus in Wistar rats. FEBS J..

[6] Koutroumanidou E., Kimbaris A., Kortsaris A., Bezirtzoglou E., Polissiou M., Charalabopoulos K., Pagonopoulou O. (2013). Increased seizure latency and decreased severity of pentylenetetrazol-induced seizures in mice after essential oil administration. Epilepsy Res. Treat..

[7] Tamber M.S., Mountz J.M. (2012). Advances in the diagnosis and treatment of epilepsy. Semin. Nucl. Med..

[8] Beyreuther B.K., Freitag J., Heers C., Krebsfänger N., Scharfenecker U., Stöhr T. (2007). Lacosamide: A review of preclinical properties. CNS Drug Rev..

[9] Bialer M., Midha K.K. (2010). Generic products of antiepileptic drugs: A perspective on bioequivalence and interchangeability. Epilepsia.

[10] Shorvon S.D., Goodridge D.M. (2013). Longitudinal cohort studies of the prognosis of epilepsy: Contribution of the National General Practice Study of Epilepsy and other studies. Brain.

[11] Gilani A.H. (2005). Trends in ethnopharmacology. J. Ethnopharmacol..

[12] Lin J.-T., Chen Y.-C., Lee Y.-C., Hou C.-W.R., Chen F.-L., Yang D.-J. (2012). Antioxidant, anti-proliferative and cyclooxygenase-2 inhibitory activities of ethanolic extracts from lemon balm (*Melissa officinalis L*.) leaves. LWT.

[13] Kähkönen M.P., Hopia A.I., Vuorela H.J., Rauha J.-P., Pihlaja K., Kujala T.S., Heinonen M. (1999). Antioxidant activity of plant extracts containing phenolic compounds. J. Agric. Food Chem..

[14] Hussain A.I. (2009). Characterization and biological activities of essential oils of some species of lamiaceae. Ph.D. Thesis.

[15] Malcolm B.J., Tallian K. (2017). Essential oil of lavender in anxiety disorders: Ready for prime time?. Ment. Health Clin..

[16] Sayorwan W., Siripornpanich V., Piriyapunyaporn T., Hongratanaworakit T., Kotchabhakdi N., Ruangrungsi N. (2012). The effects of lavender oil inhalation on emotional states, autonomic nervous system, and brain electrical activity. J. Med. Assoc. Thai..

[17] López V., Nielsen B., Solas M., Ramírez M.J., Jäger A.K. (2017). Exploring pharmacological mechanisms of lavender (Lavandula angustifolia) essential oil on central nervous system targets. Front. Pharmacol..

[18] Ayaz M., Sadiq A., Junaid M., Ullah F., Subhan F., Ahmed J. (2017). Neuroprotective and anti-aging potentials of essential oils from aromatic and medicinal plants. Front. Aging Neurosci..

[19] Justus B., Almeida V.P.d., Gonçalves M.M., Assunção D.P.d.S.F.d., Borsato D.M., Arana A.F.M., Maia B.H.L.N.S., Paula J.d.F.P.d., Budel J.M., Farago P.V. (2018). Chemical composition and biological activities of the essential oil and anatomical markers of *Lavandula dentata L*. cultivated in Brazil. Braz. Arch. Biol. Technol..

[20] Labhar A., El-Mernissi Y., Ahidar N., Zouhri A., Benamari O., Siddique F., Bashir M., Salhi A., Ahari M., Ibenmoussa S. (2024). Seasonal Variations in the Essential Oil Composition and Biological Activities of Wild *Lavandula dentata* L. Natural Product Communications.

[21] Bouyahya A., Chamkhi I., El Menyiy N., El Moudden H., Harhar H., El Idrissi Z.L., Khouchlaa A., Jouadi I., El Baaboua A., Taha D. (2023). Traditional use, phytochemistry, toxicology, and pharmacological properties of *Lavandula dentata L*.: A comprehensive review. South Afr. J. Bot..

[22] Castilla-Guerra L., Fernández-Moreno M.d.C., López-Chozas J.M., Fernández-Bolaños R. (2006). Electrolytes disturbances and seizures. Epilepsia.

[23] Raimondo J.V., Burman R.J., Katz A.A., Akerman C.J. (2015). Ion dynamics during seizures. Front. Cell. Neurosci..

[24] Abdel-Reheim E., Ahmed R., Mohamed E. (2008). Modulatory effects of pumpkin seed oil on pilocarpine–model of epilepsy in rats compared to topiramate as a common antiepileptic drug. J. Egypt. Ger. Soc. Zool. C.

[25] Gorji A., Madeja M., Straub H., Köhling R., Speckmann E.-J. (2001). Lowering of the potassium concentration induces epileptiform activity in guinea-pig hippocampal slices. Brain Res..

[26] Sirait R.H. (2019). Bahan Kuliah Fisiologi Cairan Tubuh dan Elektrolit. http://repository.uki.ac.id/2783/.

[27] Karpova L.V., Bulygina E.R., Boldyrev A.A. (2010). Different neuronal Na+/K+-ATPase isoforms are involved in diverse signaling pathways. Cell Biochem. Funct. Cell. Biochem. Modul. Act. Agents Dis..

[28] Hinterkeuser S., Schröder W., Hager G., Seifert G., Blümcke I., Elger C.E., Schramm J., Steinhäuser C. (2000). Astrocytes in the hippocampus of patients with temporal lobe epilepsy display changes in potassium conductances. Eur. J. Neurosci..

[29] Engelborghs S., D’hooge R., De Deyn P. (2000). Pathophysiology of epilepsy. Acta Neurol. Belg..

[30] Holmes G.L., Ben-Ari Y. (2001). The neurobiology and consequences of epilepsy in the developing brain. Pediatr. Res..

[31] Xie Y., Ng N.N., Safrina O.S., Ramos C.M., Ess K.C., Schwartz P.H., Smith M.A., O’Dowd D.K. (2020). Comparisons of dual isogenic human iPSC pairs identify functional alterations directly caused by an epilepsy associated SCN1A mutation. Neurobiol. Dis..

[32] Lai H.C., Jan L.Y. (2006). The distribution and targeting of neuronal voltage-gated ion channels. Nat. Rev. Neurosci..

[33] Trimmer J.S., Rhodes K.J. (2004). Localization of voltage-gated ion channels in mammalian brain. Annu. Rev. Physiol..

[34] Lorincz A., Nusser Z. (2010). Molecular identity of dendritic voltage-gated sodium channels. Science.

[35] Lipton S.A., Rosenberg P.A. (1994). Excitatory amino acids as a final common pathway for neurologic disorders. N. Engl. J. Med..

[36] Werner F.-M., Coveñas R. (2011). Classical neurotransmitters and neuropeptides involved in generalized epilepsy: A focus on antiepileptic drugs. Curr. Med. Chem..

[37] Coulter D.A., Eid T. (2012). Astrocytic regulation of glutamate homeostasis in epilepsy. Glia.

[38] Hauser R.M., Henshall D.C., Lubin F.D. (2018). The epigenetics of epilepsy and its progression. Neuroscientist.

[39] Akyuz E., Polat A.K., Eroglu E., Kullu I., Angelopoulou E., Paudel Y.N. (2021). Revisiting the role of neurotransmitters in epilepsy: An updated review. Life Sci..

[40] Freitas R.M. (2010). Lipoic acid alters δ-aminolevulinic dehydratase, glutathione peroxidase and Na+, K+-ATPase activities and glutathione-reduced levels in rat hippocampus after pilocarpine-induced seizures. Cell. Mol. Neurobiol..

[41] Silva Brum L., Emanuelli T., Souza D., Elisabetsky E. (2001). Effects of linalool on glutamate release and uptake in mouse cortical synaptosomes. Neurochem. Res..

[42] Bavarsad N.H., Bagheri S., Kourosh-Arami M., Komaki A. (2023). Aromatherapy for the brain: Lavender’s healing effect on epilepsy, depression, anxiety, migraine, and Alzheimer’s disease: A review article. Heliyon.

[43] Cifelli P., Grace A.A. (2012). Pilocarpine-induced temporal lobe epilepsy in the rat is associated with increased dopamine neuron activity. Int. J. Neuropsychopharmacol..

[44] Li J., Wang X., Xun S., Guo Q., Wang Y., Jia Y., Wang W., Wang Y., Li T., Tang T. (2022). Study of the Mechanism of Antiemetic Effect of Lavandula angustifolia Mill. Essential Oil Based on Ca^2+^/CaMKII/ERK1/2 Pathway. Drug Des. Dev. Ther..

[45] Drozdoff L., Klein E., Kiechle M., Paepke D. (2018). Use of biologically-based complementary medicine in breast and gynecological cancer patients during systemic therapy. BMC Complement. Altern. Med..

[46] Zingg J.M., Meydani M., Azzi A. (2012). α-Tocopheryl phosphate—An activated form of vitamin E important for angiogenesis and vasculogenesis?. Biofactors.

[47] Sudha K., Rao A.V., Rao A. (2001). Oxidative stress and antioxidants in epilepsy. Clin. Chim. Acta.

[48] Singh R., Pathak D.N. (1990). Lipid peroxidation and glutathione peroxidase, glutathione reductase, superoxide dismutase, catalase, and glucose-6-phosphate dehydrogenase activities in FeCl_3_-induced epileptogenic foci in the rat brain. Epilepsia.

[49] Bruce A.J., Baudry M. (1995). Oxygen free radicals in rat limbic structures after kainate-induced seizures. Free Radic. Biol. Med..

[50] Gluck M.R., Jayatilleke E., Shaw S., Rowan A.J., Haroutunian V. (2000). CNS oxidative stress associated with the kainic acid rodent model of experimental epilepsy. Epilepsy Res..

[51] Bellissimo M.I., Amado D., Abdalla D.S., Ferreira E.C., Cavalheiro E.A., da Graça Naffah-Mazzacoratti M. (2001). Superoxide dismutase, glutathione peroxidase activities and the hydroperoxide concentration are modified in the hippocampus of epileptic rats. Epilepsy Res..

[52] Ilhan A., Gurel A., Armutcu F., Kamisli S., Iraz M. (2005). Antiepileptogenic and antioxidant effects of Nigella sativa oil against pentylenetetrazol-induced kindling in mice. Neuropharmacology.

[53] Lores Arnaiz S., Travacio M., Llesuy S., Rodriguez de Lores Arnaiz G. (1998). Regional vulnerability to oxidative stress in a model of experimental epilepsy. Neurochem. Res..

[54] Svenningsen A.B., Madsen K.D., Liljefors T., Stafford G.I., van Staden J., Jäger A.K. (2006). Biflavones from Rhus species with affinity for the GABAA/benzodiazepine receptor. J. Ethnopharmacol..

[55] Kashani M.S., Tavirani M.R., Talaei S.A., Salami M. (2011). Aqueous extract of lavender (*Lavandula angustifolia*) improves the spatial performance of a rat model of Alzheimer’s disease. Neurosci. Bull..

[56] Wang D., Yuan X., Liu T., Liu L., Hu Y., Wang Z., Zheng Q. (2012). Neuroprotective activity of lavender oil on transient focal cerebral ischemia in mice. Molecules.

[57] Elisabetsky E., Marschner J., Onofre Souza D. (1995). Effects of linalool on glutamatergic system in the rat cerebral cortex. Neurochem. Res..

[58] Qneibi M., Jaradat N., Hawash M., Zaid A.N., Natsheh A.-R., Yousef R., AbuHasan Q. (2019). The neuroprotective role of *Origanum syriacum L*. and *Lavandula dentata L*. essential oils through their effects on AMPA receptors. BioMed Res. Int..

[59] Büyükokuroğlu M.E., Gepdiremen A., Hacimüftüoğlu A., Oktay M. (2003). The effects of aqueous extract of *Lavandula angustifolia* flowers in glutamate-induced neurotoxicity of cerebellar granular cell culture of rat pups. J. Ethnopharmacol..

[60] Hanrahan J.R., Chebib M., Johnston G.A. (2015). Interactions of flavonoids with ionotropic GABA receptors. Adv. Pharmacol..

[61] Jiang K., Wang W., Jin X., Wang Z., Ji Z., Meng G. (2015). Silibinin, a natural flavonoid, induces autophagy via ROS-dependent mitochondrial dysfunction and loss of ATP involving BNIP3 in human MCF7 breast cancer cells. Oncol. Rep..

[62] Leyva-López N., Gutierrez-Grijalva E.P., Ambriz-Perez D.L., Heredia J.B. (2016). Flavonoids as cytokine modulators: A possible therapy for inflammation-related diseases. Int. J. Mol. Sci..

[63] Bahr T.A., Rodriguez D., Beaumont C., Allred K. (2019). The effects of various essential oils on epilepsy and acute seizure: A systematic review. Evid. -Based Complement. Altern. Med..

[64] Costa D.A., de Oliveira G.A.L., Lima T.C., dos Santos P.S., de Sousa D.P., de Freitas R.M. (2012). Anticonvulsant and antioxidant effects of cyano-carvone and its action on acetylcholinesterase activity in mice hippocampus. Cell. Mol. Neurobiol..

[65] Somani S.J., Modi K.P., Majumdar A.S., Sadarani B.N. (2015). Phytochemicals and their potential usefulness in inflammatory bowel disease. Phytother. Res..

[66] Comalada M., Ballester I., Bailón E., Sierra S., Xaus J., Gálvez J., de Medina F.S., Zarzuelo A. (2006). Inhibition of pro-inflammatory markers in primary bone marrow-derived mouse macrophages by naturally occurring flavonoids: Analysis of the structure–activity relationship. Biochem. Pharmacol..

[67] Comalada M., Camuesco D., Sierra S., Ballester I., Xaus J., Gálvez J., Zarzuelo A. (2005). In vivo quercitrin anti-inflammatory effect involves release of quercetin, which inhibits inflammation through down-regulation of the NF-κB pathway. Eur. J. Immunol..

[68] Kita T., Asanuma M., Miyazaki I., Takeshima M. (2014). Protective effects of phytochemical antioxidants against neurotoxin-induced degeneration of dopaminergic neurons. J. Pharmacol. Sci..

[69] Raafat K., Breitinger U., Mahran L., Ayoub N., Breitinger H.-G. (2010). Synergistic inhibition of glycinergic transmission in vitro and in vivo by flavonoids and strychnine. Toxicol. Sci..

[70] Hanrahan J.R., Chebib M., Johnston G.A. (2011). Flavonoid modulation of GABAA receptors. Br. J. Pharmacol..

[71] Alexander S.P. (2006). Flavonoids as antagonists at A1 adenosine receptors. Phytother. Res. Int. J. Devoted Pharmacol. Toxicol. Eval. Nat. Prod. Deriv..

[72] Algieri F., Rodriguez-Nogales A., Vezza T., Garrido-Mesa J., Garrido-Mesa N., Utrilla M.P., González-Tejero M.R., Casares-Porcel M., Molero-Mesa J., del Mar Contreras M. (2016). Anti-inflammatory activity of hydroalcoholic extracts of *Lavandula dentata L*. and *Lavandula stoechas* L. J. Ethnopharmacol..

[73] Guimarães A.G., Quintans J.S., Quintans-Júnior L.J. (2013). Monoterpenes with analgesic activity—A systematic review. Phytother. Res..

[74] Guilhon C.C., Raymundo L.J., Alviano D.S., Blank A.F., Arrigoni-Blank M.F., Matheus M.E., Cavalcanti S.C., Alviano C.S., Fernandes P.D. (2011). Characterisation of the anti-inflammatory and antinociceptive activities and the mechanism of the action of *Lippia gracilis* essential oil. J. Ethnopharmacol..

[75] Miyazawa M., Yamafuji C. (2005). Inhibition of acetylcholinesterase activity by bicyclic monoterpenoids. J. Agric. Food Chem..

[76] Quintans-Júnior L.J., Melo M.S., De Sousa D.P., Araujo A., Onofre A., Gelain D.P., Gonçalves J., Araújo D., Almeida J., Bonjardim L.R. (2010). Antinociceptive effects of citronellal in formalin-, capsaicin-, and glutamate-induced orofacial nociception in rodents and its action on nerve excitability. J. Oral Facial Pain Headache.

[77] Felipe C.F.B., Albuquerque A.M.S., de Pontes J.L.X., de Melo J.Í.V., Rodrigues T.C.M.L., de Sousa A.M.P., Monteiro Á.B., Ribeiro A.E.d.S., Lopes J.P., de Menezes I.R.A. (2019). Comparative study of alpha-and beta-pinene effect on PTZ-induced convulsions in mice. Fundam. Clin. Pharmacol..

[78] Skalicka-Woźniak K., Walasek M., Aljarba T.M., Stapleton P., Gibbons S., Xiao J., Łuszczki J.J. (2018). The anticonvulsant and anti-plasmid conjugation potential of *Thymus vulgaris* chemistry: An in vivo murine and in vitro study. Food Chem. Toxicol..

[79] Jiang J., Shen Y.Y., Li J., Lin Y.H., Luo C.X., Zhu D.Y. (2015). (+)-Borneol alleviates mechanical hyperalgesia in models of chronic inflammatory and neuropathic pain in mice. Eur. J. Pharmacol..

[80] Nguyen P.T.T., Jang S.H., Rijal S., Park S.J., Han S.K. (2020). Inhibitory actions of borneol on the substantia gelatinosa neurons of the trigeminal subnucleus caudalis in mice. Korean J. Physiol. Pharmacol. Off. J. Korean Physiol. Soc. Korean Soc. Pharmacol..

[81] Conforti F., Statti G.A., Tundis R., Loizzo M.R., Menichini F. (2007). In vitro activities of *Citrus medica L*. cv. Diamante (Diamante citron) relevant to treatment of diabetes and Alzheimer’s disease. Phytother. Res. Int. J. Devoted Pharmacol. Toxicol. Eval. Nat. Prod. Deriv..

[82] Boiangiu R.S., Brinza I., Hancianu M., Erdogan Orhan I., Eren G., Gündüz E., Ertas H., Hritcu L., Cioanca O. (2020). Cognitive facilitation and antioxidant effects of an essential oil mix on scopolamine-induced amnesia in rats: Molecular modeling of in vitro and in vivo approaches. Molecules.

[83] Ku C.-M., Lin J.-Y. (2013). Anti-inflammatory effects of 27 selected terpenoid compounds tested through modulating Th1/Th2 cytokine secretion profiles using murine primary splenocytes. Food Chem..

[84] Milanos S., Elsharif S.A., Janzen D., Buettner A., Villmann C. (2017). Metabolic products of linalool and modulation of GABAA receptors. Front. Chem..

[85] Souto-Maior F.N., Fonsêca D.V.d., Salgado P.R.R., Monte L.d.O., de Sousa D.P., de Almeida R.N. (2017). Antinociceptive and anticonvulsant effects of the monoterpene linalool oxide. Pharm. Biol..

[86] El Alaoui C., Chemin J., Fechtali T., Lory P. (2017). Modulation of T-type Ca2+ channels by Lavender and Rosemary extracts. PLoS ONE.

[87] Wei F., Yan L.-M., Su T., He N., Lin Z.-J., Wang J., Shi Y.-W., Yi Y.-H., Liao W.-P. (2017). Ion channel genes and epilepsy: Functional alteration, pathogenic potential, and mechanism of epilepsy. Neurosci. Bull..

[88] Li Z.M., Liu X.X., Li C., Wei Z.C., Shi Y., Song H.Y., Chen X., Zhang Y., Li J.W., Zhu R.F. (2022). Decreased synapse-associated proteins are associated with the onset of epileptic memory impairment in endothelial *CDK5*-deficient mice. MedComm.

[89] De Simoni M.G., Perego C., Ravizza T., Moneta D., Conti M., Marchesi F., De Luigi A., Garattini S., Vezzani A. (2000). Inflammatory cytokines and related genes are induced in the rat hippocampus by limbic status epilepticus. Eur. J. Neurosci..

[90] Cacheaux L.P., Ivens S., David Y., Lakhter A.J., Bar-Klein G., Shapira M., Heinemann U., Friedman A., Kaufer D. (2009). Transcriptome profiling reveals TGF-β signaling involvement in epileptogenesis. J. Neurosci..

[91] de Oliveira T.M., de Carvalho R.B.F., da Costa I.H.F., de Oliveira G.A.L., de Souza A.A., de Lima S.G., de Freitas R.M. (2015). Evaluation of p-cymene, a natural antioxidant. Pharm. Biol..

[92] Dringen R., Gutterer J.M., Hirrlinger J. (2000). Glutathione metabolism in brain: Metabolic interaction between astrocytes and neurons in the defense against reactive oxygen species. Eur. J. Biochem..

[93] Horváthová E., Slameňová D., Maršálková L., Šramková M., Wsólová L. (2009). Effects of borneol on the level of DNA damage induced in primary rat hepatocytes and testicular cells by hydrogen peroxide. Food Chem. Toxicol..

[94] Liu R., Zhang L., Lan X., Li L., Zhang T.-T., Sun J.-H., Du G.-H. (2011). Protection by borneol on cortical neurons against oxygen-glucose deprivation/reperfusion: Involvement of anti-oxidation and anti-inflammation through nuclear transcription factor κappaB signaling pathway. Neuroscience.

[95] Tambe R., Jain P., Patil S., Ghumatkar P., Sathaye S. (2016). Antiepileptogenic effects of borneol in pentylenetetrazole-induced kindling in mice. Naunyn-Schmiedeb. Arch. Pharmacol..

[96] Wu H.-Y., Tang Y., Gao L.-Y., Sun W.-X., Hua Y., Yang S.-B., Zhang Z.-P., Liao G.-Y., Zhou Q.-G., Luo C.-X. (2014). The synergetic effect of edaravone and borneol in the rat model of ischemic stroke. Eur. J. Pharmacol..

[97] Delgado-Marín L., Sánchez-Borzone M., García D.A. (2017). Neuroprotective effects of gabaergic phenols correlated with their pharmacological and antioxidant properties. Life Sci..

[98] Aydın E., Turkez H., Tasdemir S., Hacımuftuoglu F. (2017). Anticancer, antioxidant and cytotoxic potential of thymol in vitro brain tumor cell model. Cent. Nerv. Syst. Agents Med. Chem..

[99] Venu S., Naik D., Sarkar S., Aravind U.K., Nijamudheen A., Aravindakumar C. (2013). Oxidation reactions of thymol: A pulse radiolysis and theoretical study. J. Phys. Chem. A.

[100] Nagoor Meeran M.F., Stanely Mainzen Prince P. (2012). Protective effects of thymol on altered plasma lipid peroxidation and nonenzymic antioxidants in isoproterenol-induced myocardial infarcted rats. J. Biochem. Mol. Toxicol..

[101] Asadbegi M., Komaki A., Salehi I., Yaghmaei P., Ebrahim-Habibi A., Shahidi S., Sarihi A., Asl S.S., Golipoor Z. (2018). Effects of thymol on amyloid-β-induced impairments in hippocampal synaptic plasticity in rats fed a high-fat diet. Brain Res. Bull..

[102] Rabidas S.S., Prakash C., Tyagi J., Suryavanshi J., Kumar P., Bhattacharya J., Sharma D. (2023). A comprehensive review on anti-inflammatory response of flavonoids in experimentally-induced epileptic seizures. Brain Sci..

[103] Ngaibi J., Kandeda A.K., Nguezeye Y., Wangbara T.A., Gaoudji L., Taiwe G.S., Bum E.N. (2024). Antiepileptic and anti-inflammatory effects of *Lippia multiflora* moldenke (Verbenaceae) in mice model of chronic temporal lobe epilepsy induced by pilocarpine. Heliyon.

[104] Azimi S., Rahmati B., Roghani M., Sedighnejad L. (2021). The effects of aerial parts hydroalcoholic extract of *Lavandula dentata* in the pilocarpine rat model of temporal lobe epilepsy. Daneshvar Med..

[105] Houssni A., Youness E.A., Meryem J., El Bouazzi O., Aimad A., Karima M. (2024). Extract and Fixed Oil of Moroccan *Lavandula dentata* Aerial Parts as Potential Antioxidants. Trop. J. Nat. Prod. Res..

[106] Zhang Y., Hu X., Zou L.-Q. (2024). Flavonoids as therapeutic agents for epilepsy: Unveiling anti-inflammatory and antioxidant pathways for novel treatments. Front. Pharmacol..

[107] Liu W., Ge T., Pan Z., Leng Y., Lv J., Li B. (2017). The effects of herbal medicine on epilepsy. Oncotarget.

[108] Gholijani N., Amirghofran Z. (2016). Effects of thymol and carvacrol on T-helper cell subset cytokines and their main transcription factors in ovalbumin-immunized mice. J. Immunotoxicol..

[109] Mahmoodi M., Amiri H., Ayoobi F., Rahmani M., Taghipour Z., Ghavamabadi R.T., Jafarzadeh A., Sankian M. (2019). Carvacrol ameliorates experimental autoimmune encephalomyelitis through modulating pro-and anti-inflammatory cytokines. Life Sci..

[110] Vieira É.L., de Oliveira G.N., Lessa J.M.K., Gonçalves A.P., Sander J.W., Cendes F., Teixeira A.L. (2015). Interleukin-1β plasma levels are associated with depression in temporal lobe epilepsy. Epilepsy Behav..

[111] Meng F., Yao L. (2020). The role of inflammation in epileptogenesis. Acta Epileptol..

[112] Lee Y.B., Nagai A., Kim S.U. (2002). Cytokines, chemokines, and cytokine receptors in human microglia. J. Neurosci. Res..

[113] Wang Y., Wang D., Guo D. (2015). Interictal cytokine levels were correlated to seizure severity of epileptic patients: A retrospective study on 1218 epileptic patients. J. Transl. Med..

[114] Sinha S., Patil S., Jayalekshmy V., Satishchandra P. (2008). Do cytokines have any role in epilepsy?. Epilepsy Res..

[115] Mao L.Y., Ding J., Peng W.F., Ma Y., Zhang Y.H., Fan W., Wang X. (2013). Interictal interleukin-17 A levels are elevated and correlate with seizure severity of epilepsy patients. Epilepsia.

[116] Pernhorst K., Herms S., Hoffmann P., Cichon S., Schulz H., Sander T., Schoch S., Becker A.J., Grote A. (2013). TLR4, ATF-3 and IL8 inflammation mediator expression correlates with seizure frequency in human epileptic brain tissue. Seizure.

[117] Sonmez F.M., Serin H.M., Alver A., Aliyazicioglu R., Cansu A., Can G., Zaman D. (2013). Blood levels of cytokines in children with idiopathic partial and generalized epilepsy. Seizure.

[118] Lopes A.M., Buosi P., de Mattos Farina B., Fernandes-Ferreira R., Oliveira-Brancati C.I.F., Martins D.P., da Silva D.G.H., Chaves N.A., Marques L.H.N., de Araújo Filho G.M. (2023). Serum levels of oxidative stress biomarkers is changed in pharmacoresistant mesial temporal lobe epilepsy patients with or without psychiatric disorders. Brain Disord..

[119] Parsons A.L., Bucknor E.M., Castroflorio E., Soares T.R., Oliver P.L., Rial D. (2022). The interconnected mechanisms of oxidative stress and neuroinflammation in epilepsy. Antioxidants.

[120] Fabisiak T., Patel M. (2022). Crosstalk between neuroinflammation and oxidative stress in epilepsy. Front. Cell Dev. Biol..

[121] Wang L., Liang Q., Lin A., Wu Y., Min H., Song S., Wang Y., Wang H., Yi L., Gao Q. (2019). Borneol alleviates brain injury in sepsis mice by blocking neuronal effect of endotoxin. Life Sci..

[122] Bauer J., Vezzani A., Bien C.G. (2012). Epileptic encephalitis: The role of the innate and adaptive immune system. Brain Pathol..

[123] Zhou Z., Peng X., Insolera R., Fink D.J., Mata M. (2009). Interleukin-10 provides direct trophic support to neurons. J. Neurochem..

[124] Ashkenazi A., Dixit V.M. (1998). Death receptors: Signaling and modulation. Science.

[125] Pahan K., Khan M., Singh I. (2000). Interleukin-10 and interleukin-13 inhibit proinflammatory cytokine-induced ceramide production through the activation of phosphatidylinositol 3-kinase. J. Neurochem..

[126] Bachis A., Colangelo A.M., Vicini S., Doe P.P., De Bernardi M.A., Brooker G., Mocchetti I. (2001). Interleukin-10 prevents glutamate-mediated cerebellar granule cell death by blocking caspase-3-like activity. J. Neurosci..

[127] Grilli M., Barbieri I., Basudev H., Brusa R., Casati C., Lozza G., Ongini E. (2000). Interleukin-10 modulates neuronal threshold of vulnerability to ischaemic damage. Eur. J. Neurosci..

[128] Strle K., Zhou J.-H., Broussard S.R., Venters H.D., Johnson R.W., Freund G.G., Dantzer R., Kelley K.W. (2002). IL-10 promotes survival of microglia without activating Akt. J. Neuroimmunol..

[129] Brodie C. (1996). Differential effects of Th1 and Th2 derived cytokines on NGF synthesis by mouse astrocytes. FEBS Lett..

[130] Gao F., Gao Y., Zhang S.J., Zhe X., Meng F.l., Qian H., Zhang B., Li Y.j. (2017). Alteration of plasma cytokines in patients with active epilepsy. Acta Neurol. Scand..

[131] LM W. (2007). Maintenance of the Foxp3-dependent developmental program in mature regulatory T cells requires continued expression of Foxp3. Nat. Immunol..

[132] Wang J., Lin Z.-J., Liu L., Xu H.-Q., Shi Y.-W., Yi Y.-H., He N., Liao W.-P. (2017). Epilepsy-associated genes. Seizure.

[133] McDonald D.R. (2012). TH17 deficiency in human disease. J. Allergy Clin. Immunol..

[134] Faridzadeh A., Salimi Y., Ghasemirad H., Kargar M., Rashtchian A., Mahmoudvand G., Karimi M.A., Zerangian N., Jahani N., Masoudi A. (2022). Neuroprotective Potential of Aromatic Herbs: Rosemary, Sage, and Lavender. Front. Neurosci..

[135] Jeffery K.J. (2018). The hippocampus: From memory, to map, to memory map. Trends Neurosci..

[136] Brandt C., Potschka H., Löscher W., Ebert U. (2003). N-methyl-D-aspartate receptor blockade after status epilepticus protects against limbic brain damage but not against epilepsy in the kainate model of temporal lobe epilepsy. Neuroscience.

[137] Bui A.D., Nguyen T.M., Limouse C., Kim H.K., Szabo G.G., Felong S., Maroso M., Soltesz I. (2018). Dentate gyrus mossy cells control spontaneous convulsive seizures and spatial memory. Science.

[138] Nikonenko A.G., Radenovic L., Andjus P.R., Skibo G.G. (2009). Structural features of ischemic damage in the hippocampus. Anat. Rec..

[139] Davies N. (1990). Gas chromatographic retention indices of monoterpenes and sesquiterpenes on methyl silicon and Carbowax 20M phases. J. Chromatogr. A.

[140] Adams R.P. (2017). Identification of Essential Oil Components by Gas Chromatography/Mass Spectrometry.

[141] Blažeković B., Yang W., Wang Y., Li C., Kindl M., Pepeljnjak S., Vladimir-Knežević S. (2018). Chemical composition, antimicrobial and antioxidant activities of essential oils of *Lavandula* × *intermedia* ‘Budrovka’and *L. angustifolia* cultivated in Croatia. Ind. Crops Prod..

[142] Turski L., Ikonomidou C., Turski W.A., Bortolotto Z.A., Cavalheiro E.A. (1989). Cholinergic mechanisms and epileptogenesis. The seizures induced by pilocarpine: A novel experimental model of intractable epilepsy. Synapse.

[143] Abdel-Reheim E.S. (2009). Physiological and biochemical studies on the melatonin effect on the fertility of epileptic rats. J. Egyp. Ger. Soci. Zool..

[144] Raza M., Dhariwal M.A., Ageel A.M., Qureshi S. (1996). Evaluation of the antiinflammatory activity of sodium valproate in rats and mice. Gen. Pharmacol. Vasc. Syst..

[145] Ortinski P., Meador K.J. (2004). Cognitive side effects of antiepileptic drugs. Epilepsy Behav..

[146] Abd El-Hameed A.M., Abuelsaad A.S., Khalil A. (2021). Bee venom acupuncture therapy ameliorates neuroinflammatory alterations in a pilocarpine-induced epilepticus model. Metab. Brain Dis..

[147] Bradford M.M. (1976). A rapid and sensitive method for the quantitation of microgram quantities of protein utilizing the principle of protein-dye binding. Anal. Biochem..

[148] Uchiyama M., Mihara M. (1978). Determination of malonaldehyde precursor in tissues by thiobarbituric acid test. Anal. Biochem..

[149] Feelisch M., Noack E. (1987). Nitric oxide (NO) formation from nitrovasodilators occurs independently of hemoglobin or non-heme iron. Eur. J. Pharmacol..

[150] Sinet P., Michelson A., Bazin A., Lejeune J., Jerome H. (1975). Increase in glutathione peroxidase activity in erythrocytes from trisomy 21 subjects. Biochem. Biophys. Res. Commun..

[151] Nishikimi M., Rao N.A., Yagi K. (1972). The occurrence of superoxide anion in the reaction of reduced phenazine methosulfate and molecular oxygen. Biochem. Biophys. Res. Commun..

[152] Delic D., Gailus N., Vohr H.-W., Dkhil M., Al-Quraishy S., Wunderlich F. (2010). Testosterone-induced permanent changes of hepatic gene expression in female mice sustained during Plasmodium chabaudi malaria infection. J. Mol. Endocrinol..

[153] Bleda S., de Haro J., Varela C., Ferruelo A., Acin F. (2016). Elevated levels of triglycerides and vldl-cholesterol provoke activation of nlrp1 inflammasome in endothelial cells. Int. J. Cardiol..

[154] Bancroft J.D., Gamble M. (2008). Theory and Practice of Histological Techniques.

[155] Abdel-Latif R.G., Heeba G.H., Taye A., Khalifa M.M. (2018). Lixisenatide, a novel GLP-1 analog, protects against cerebral ischemia/reperfusion injury in diabetic rats. Naunyn-Schmiedeb. Arch. Pharmacol..

[156] Lopès A., Cassé A.H., Billard E., Boulcourt-Sambou E., Roche G., Larois C., Barnich N., Naimi S., Bonnet M., Dumas B. (2018). Deciphering the immune microenvironment of a tissue by digital imaging and cognition network. Sci. Rep..

